# The Janus Face of PAMAM Dendrimers Used to Potentially Cure Nonenzymatic Modifications of Biomacromolecules in Metabolic Disorders—A Critical Review of the Pros and Cons

**DOI:** 10.3390/molecules181113769

**Published:** 2013-11-07

**Authors:** Magdalena Labieniec-Watala, Kamil Karolczak, Karolina Siewiera, Cezary Watala

**Affiliations:** 1 Department of Thermobiology, Faculty of Biology and Environmental Protection, University of Lodz, Pomorska 141/143, Lodz 90-236, Poland; 2 Department of Haemostasis and Haemostatic Disorders, Faculty of Health Sciences, Medical University of Lodz, Zeromskiego 113, Lodz 90-549, Poland; E-Mails: kamilkarolczak@gmail.com (K.K.); ksiewiera@gmail.com (K.S.); cezary.watala@umed.lodz.pl (C.W.)

**Keywords:** PAMAM dendrimers, diabetes mellitus, hyperglycaemia, mitochondria function, bioenergetics, non-enzymatic glycosylation, diabetic markers, *in vivo* study

## Abstract

Diabetes mellitus, which is characterised by high blood glucose levels and the burden of various macrovascular and microvascular complications, is a cause of much human suffering across the globe. While the use of exogenous insulin and other medications can control and sometimes prevent various diabetes-associated sequelae, numerous diabetic complications are still commonly encountered in diabetic patients. Therefore, there is a strong need for safe and effective antihyperglycaemic agents that provide an alternative or compounding option for the treatment of diabetes. In recent years, amino-terminated poly(amido)amine (PAMAM) dendrimers (G2, G3 and G4) have attracted attention due to their protective value as anti-glycation and anti-carbonylation agents that can be used to limit the nonenzymatic modifications of biomacromolecules. The focus of this review is to present a detailed survey of our own data, as well as of the available literature regarding the toxicity, pharmacological properties and overall usefulness of PAMAM dendrimers. This presentation pays particular and primary attention to their therapeutic use in poorly controlled diabetes and its complications, but also in other conditions, such as Alzheimer’s disease, in which such nonenzymatic modifications may underlie the pathophysiological mechanisms. The impact of dendrimer administration on the overall survival of diabetic animals and on glycosylation, glycoxidation, the brain-blood barrier and cellular bioenergetics are demonstrated. Finally, we critically discuss the potential advantages and disadvantages accompanying the use of PAMAM dendrimers in the treatment of metabolic impairments that occur under conditions of chronic hyperglycaemia.

## 1. Introduction

In 1984, in their article titled “New class of polymers: STARBURST-dendritic macromolecules”, Donald Tomalia and co-authors wrote: “This paper describes the first synthesis of a new class of topological macromolecules, which we refer to as “starburst polymers”. The fundamental building blocks of this new polymer class are referred to as “dendrimers” [1]. More than two decades later, in 2009, in their paper “New insight into the interactions between dendrimers and surfactants…” Cheng and co-authors proposed a new drug formulation based on dendrimer compounds [2]. Presently, publications with titles beginning with the words “novel” or “new” and concerning the use of amino-terminated poly(amido)amine (PAMAM) dendrimers in chemical and biological studies can still be found [3]. Clearly, current research on the action of PAMAM dendrimers in biological systems and their possible applications still emphasises the various novel attributes of these nanoparticles. Despite the passage of time, the number of areas in biomedicine and related fields in which research on dendrimer applications is being pursued has continued to grow. Indeed, the special architecture and physiological behaviour of dendrimers, especially PAMAMs, makes them suitable for numerous applications in different fields of science.

A survey of the current literature shows that many potential applications of various dendrimers in biomedicine have been extensively explored, and the number of reports on these applications is untold. Therefore, in this review, which is a natural continuation of our earlier review [4] and some original papers [5,6,7], we pass over basic information about dendrimer structure and the details concerning their synthesis and biomedical properties. The reader is referred to the aforementioned papers and to other excellent literature on the chemistry and possible implementations of PAMAM dendrimers [8,9,10,11,12].

**Figure 1:**
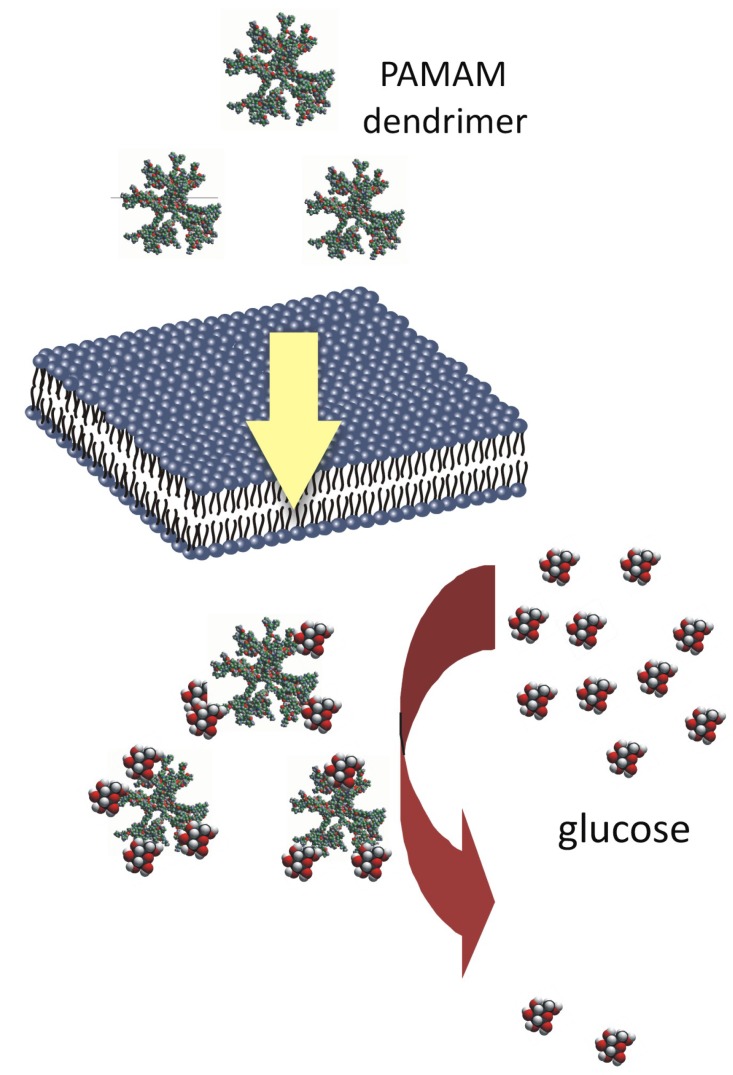
Schematic diagram depicting the basis for the use of full-generation PAMAM dendrimers as “scavengers” of excessive glucose.

Herein, we present and develop the idea of using PAMAM dendrimers (G2, G3 and G4) as a novel strategy for the treatment of metabolic impairments associated with nonenzymatic modifications of biomacromolecules, with a particular focus on diabetes mellitus and related pathophysiological states. In 2007, we initiated the first *in vivo* experiment designed to test the hypothesis that 4th-generation PAMAM dendrimers can effectively limit the consequences of long-lasting diabetes in rats. This cationic agent, bearing sixty-four amine groups on its surface, has shown potent anti-glycaemic activity, lowering not only excessive plasma glucose levels *per se* but also decreasing the concentration of products of the nonenzymatic modifications of macromolecules that are formed as a consequence of glucose and carbonyl stress (Figure 1). The results of this work motivated us to carry out further work aimed at finding the best design and formula for using PAMAMs in diabetes therapy. In that regard, we propose a novel course of research directed at searching for the biological activity of these compounds. Thus, the overall goal of our studies is to identify the mechanism(s) of action of PAMAM dendrimers in living organisms and to explore their side effects in these organisms, as well as to select the correct dose or duration of treatment with PAMAMs that effectively reduces the complications caused by diabetes-associated metabolic disorders. We are now prepared to critically review the results of these studies and to discuss opportunities regarding the use of cationic PAMAMs in this area of biomedical interest.

In the context of present knowledge concerning diabetes, a severe and complex metabolic disorder, our attention has turned to the use of amine-terminated PAMAM dendrimers as potentially functional therapeutic tools in the prevention or alleviation of diabetes-associated metabolic complications. The present review was conducted primarily to provide a better understanding of the possible hypoglycaemic action of these dendrimers, but it is also intended to describe both the putative health benefits and the possible drawbacks of the use of these compounds. We consider it of the utmost importance to critically evaluate the available literature on the progress and setbacks concerning the use of PAMAM dendrimers in the field of biomedicine. Because the practical medical implementations of PAMAMs remain unclear, we have attempted to discuss the Janus-faced nature of these dendrimers in the context of experimental design and with attention to the possibilities for data misinterpretation.

## 2. PAMAMs as Scavengers of Excessive Glucose in Experimental Diabetes

It has been clearly shown that due to the presence of multiple terminal groups on the exterior of the molecule, dendrimers provide an excellent platform for the attachment and presentation of cell-specific targeting groups, solubility modifiers, stealth moieties that reduce immunological interactions and imaging tags. Dendrimers would hence appear to be a class of compounds that offer unlimited possibilities for use in various fields of biology, chemistry, medicine and nanotechnology. Therefore, the suggestion that dendrimers might be useful in the prevention of complications of diabetes should not be surprising.

Historically, PAMAM-based dendrimers with built-in amine functionalities on their surfaces were first used as scaffolds for the attachment of sugars. The very first example of saccharide-substituted PAMAM dendrimers was described by Aoi and co-workers, who described the synthesis of so-called “sugar balls” *via* amide bond formation starting from sugar lactones [13]. Based on the ‘sugar-binding’ properties of full-generation PAMAM dendrimers, we propose to use them in a way that is unique in the field of biomedicine.

In recent years, considerable progress has been made in the development of a new scientific field of interest involving the development and use of “glycodendrimers” and “glycopeptide dendrimers”. A review of the literature on these types of dendrimers, which is growing at a considerable rate, makes the analogy between the non-enzymatically N-glucosylated (glycated) dendrimers obtained in our studies and chemically synthesised dendrimers with attached saccharide moieties more apparent. Thus, although the two types of compounds were developed using different experimental approaches, the final products may be similar. In the first case, the aim is to derive the glycodendrimer as a product of a long-lasting non-enzymatic reaction between free dendrimer molecules and abundant free glucose, when the two are present together in a test tube or in an organism. In the other case, “glycodendrimers” are obtained *via* more-or-less complicated synthetic procedures; the latter thus possess more controllable structures and more predictable properties than dendrimers that are glycosylated in non-enzymatic reactions [14]. Another very important difference between these two classes of dendrimers is their potential for direct application. Chemically synthesised glycodendrimers, as referred to in the literature, are being used in many areas of medicine and biotechnology as polymeric anti-cancer nanomedicines and nano-carriers and as crosslinking agents, modulators of surface charge and primary components in scaffolds that mimic natural matrices [10]. On the other hand, the term “glycodendrimers”, which we have applied to the compounds studied in our laboratory, refers to the final products of non-catalysed reactions between plain dendrimers and glucose. We use the term “N-glycosylated dendrimers” or “glycated dendrimers” to indicate dendrimers that are formed as final products in the process of non-enzymatic glycosylation, which has been shown to be a driving force for protein glycation. In a previous report, we demonstrated in an *in vitro* study that plain PAMAM dendrimers react with glucose molecules to form stable bonds [7]. Based on these findings, we believe that the nonenzymatic reaction between glucose and the terminal amine groups of dendrimers may underlie the formation of N-glycosylated (“glycated”) dendrimers and that in this way PAMAM dendrimers can act as glucose scavengers during hyperglycaemia, finally resulting in reduced protein glycation and glycoxidation. Hence, contrary to other papers concerning the dendrimers generated in the course of well-controlled chemical synthesis, we propose a quite different mechanism for obtaining glycosylated dendrimers *via* spontaneous reactions that can occur on the dendrimer surface under conditions of free glucose overload. The glycodendrimers formed in this way are only the final by-products of nonenzymatic N-glycosylation and are not obtained as a result of a designed and oriented chemical synthesis.

### 2.1. Survey of the Use of PAMAM Dendrimers as Hypoglycaemic Agents in an Animal Model of Diabetes

The hypothesis that PAMAM dendrimers, by scavenging of glucose excess, are able to improve the lifespan and other parameters of individuals suffering from diabetes was tested by evaluating survival and selected biochemical parameters in an experimental model of diabetes. First, rats with streptozotocin-induced diabetes received PAMAM G4 dendrimers for 60 days; then, based on the accumulated data [5], dendrimers of the generations 2 and 3 were also administered using the same experimental design. The dosages were selected on the basis of our own observations (G4: 7.26 mg/kg ⇒ 0.511 μmol/kg/day ⇒ 33 μmol NH_2_/kg/day; G3: 20 mg/kg/day ⇒ 2.895 μmol/kg/day ⇒ 93 μmol NH_2_/kg/day and G2: 40 mg/kg/day ⇒ 12.285 μmol/kg/day ⇒ 197 μmol NH_2_/kg/day); these dosages are relatively low compared to those described in other *in vivo* reports as toxic or cytotoxic [5,15,16,17,18]. In most cases, the dendrimers were given by intraperitoneal injection, although other routes of administration were also tested. Methanol was used as a solvent for the dendrimers (0.501 µmol per a single injection/day/rat; the cumulated injected amount over the two-month treatment was 30.4 µmol). We made this choice for two main reasons: the very good solubility and excellent long-term stability of PAMAM dendrimers in methanol. To determine whether PAMAM dendrimers demonstrate hypoglycaemising effects, we monitored the following products of diabetes-associated severe hyperglycaemia, protein glycation and oxidation: non-fasting blood glycaemia, glycated haemoglobin (HbA_1c_), advanced glycation end products (AGEs), and advanced oxidation protein products (AOPP). The overall effectiveness of the hypoglycaemising effects of PAMAM dendrimers (comprehensive score, CS: a lower absolute CS value reflects a higher effectiveness of the dendrimer in reducing hyperglycaemia, glycation and glycoxidation) was evaluated using the idea of the calculus invented and reported earlier [5,19]. Several other routinely monitored biochemical parameters of whole blood (Ala and Asp aminotransferases, alkaline phosphatase, serum lipids, C-reactive protein, plasma protein fractions, urea and uric acid, creatinine, liver tests), as well as parameters characterising the bioenergetics of hepatocyte and cardiomyocyte mitochondria including ATP production, electron chain transport activity, and the integrity of mitochondrial membranes were also evaluated.

**Figure 2:**
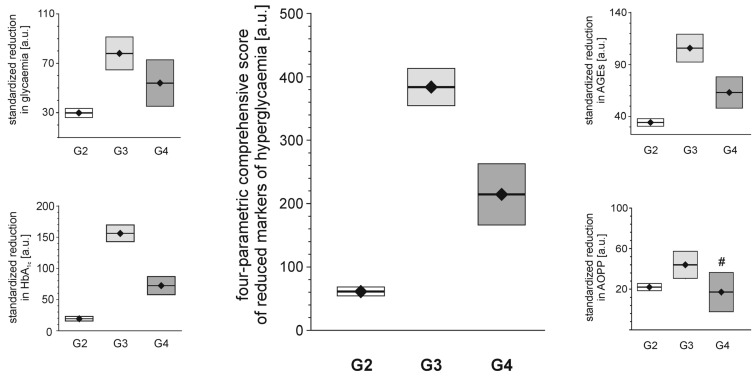
Reduction in markers of severe hyperglycaemia in streptozotocin-diabetic rats after administration of PAMAM dendrimers. The data, which are presented as the mean ± 95%CI (n = 5–19), represent standardised reductions in hyperglycaemia/oxidation markers in Sprague-Dawley rats that received PAMAM dendrimers intraperitoneally for 60 days. All the PAMAM-induced reductions except those marked ^#^ were significant (*p* < 0.0001, single sample t test). The standardised reductions after administration of PAMAM G2, G3 and G4 (16, 32 and 64 surface primary amino groups, respectively) are expressed per μmol of dendrimer surface NH_2_ per kg body weight per day (for more details, see the text). CS = [Std (x/SD): glycaemia_PAMAM_ − 31.64] + [Std (x/SD): HbA_1c__(PAMAM)_ − 8.36] + [Std (x/SD): AGEs_PAMAM_ − 9.26] + [Std (x/SD): AOPP_PAMAM_ − 12.76], where[glycaemia_PAMAM_ − 31.64], [HbA_1c__(PAMAM)_ − 8.36], [AGEs_PAMAM_ − 9.26] and [AOPP_PAMAM_ − 12.76] are the calculated differences between the observed values and the accepted threshold values (for the vehicle) [20]. The significance of the differences between PAMAM dendrimers were: *p* < 0.01 or less, μ_G2_ ≠ μ_G3_, for glycaemia, AGEs, *p* < 0.05 or less, μ_G2_ ≠ μ_G3_ ≠ μ_G4_ for HbA_1c_, AOPP, CS.

**Figure 3:**
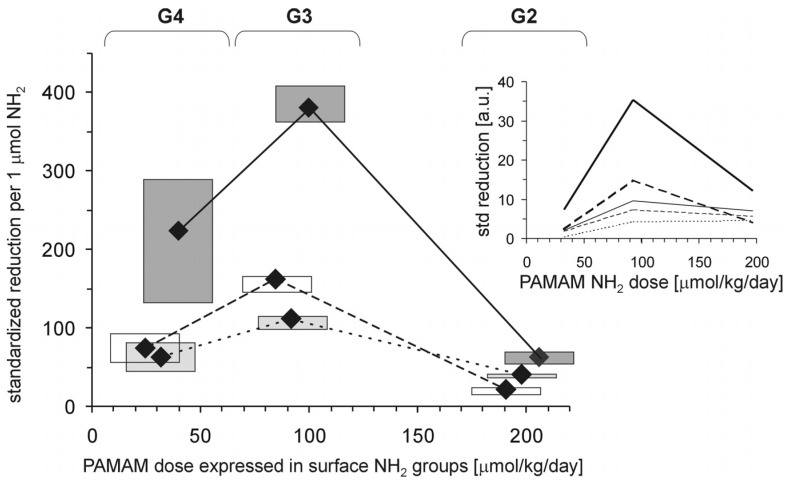
Relationship between the concentration of PAMAM surface primary amino groups and dendrimer-induced reduction in markers of prolonged hyperglycaemia in streptozotocin-diabetic rats. The data, which are presented as median and interquartile range (n = 5–19), represent standardised reductions in hyperglycamia/oxidation markers in Sprague-Dawley rats treated intraperitoneally with PAMAM dendrimers and are presented in arbitrary units [a.u.] or expressed per μmol NH_2_ per kg body weight per day (HbA_1c_: thick dashed line; AGEs: thick dotted line; and CS: thick solid line). For more details, see Figure 2 and the text. Insert: non-fasting glycaemia: thin dashed line; HbA_1c_: thick dashed line; AGEs: thin solid line; AOPP: thin dotted line; and CS: thick solid line. The significance of the differences between PAMAM dendrimers was: μ_G2_ ≠ μ_G3_ ≠ μ_G4_, *p* < 0.005 or less for all variables.

Each of the tested full-generation PAMAM dendrimers (G2, G3 and G4) significantly reduced the major hallmarks of severe hyperglycaemia as revealed by four monitored parameters: blood glycaemia, HbA_1c_, plasma AGEs and AOPP (Figure 2). Here, we can compare the effectiveness of three dendrimers differing in the number of surface primary amino groups: PAMAM G2 (16 amino groups), G3 (32 amino groups) and G4 (64 amino groups). The data show that the observed reductions in all the analysed hallmarks of severe hyperglycaemia were the greatest for PAMAM G3 and the lowest for PAMAM G2. The best discrimination between the dendrimers was shown by the four-parametric comprehensive score (CS) (Figure 2). When the effects were standardised to the so-called “partial effectiveness” of dendrimers, attributed to the single primary amino group on the PAMAM’s surface, their effects as hypoglycaemising agents were also unequal: the greatest reduction occurred after administration of PAMAM G3, which has an intermediate density of amino groups on its surface, while lower effectiveness of PAMAM G2 and PAMAM G4, which possess the lowest and the highest number of surface amino groups, respectively, was observed (Figure 3). This trend was valid for all the monitored hallmarks of hyperglycaemia, but was the most distinct for HbA_1c_, AGEs and CS (Figure 3). Overall, it should be emphasised that the number of surface amino groups (the “dose of amino groups” administered per kg of body weight) was not the most important determinant of the activity of the dendrimer in reducing severe hyperglycaemia and its metabolic consequences. Another issue of crucial practical relevance is how to reach a compromise between the desired effects and the toxicity of PAMAM dendrimers (see subsection 2.1.2).

In general, it was found that administration of PAMAM dendrimers significantly reduces hyperglycaemia and levels of glycation/glycoxidation, regardless of the generation of dendrimers used. The data clearly indicate that dendrimers can be considered hypoglycaemising/anti-glycation agents, although the monitored parameters still remain far from being normalised to physiological levels. Hence, in our opinion, PAMAM dendrimers should be regarded as hypoglycaemising agents rather than typical “anti-diabetic” agents [5,6,19,21].

#### 2.1.1. Two Faces of PAMAMs in Experimental Diabetes: Beneficial Therapeutic Effects and Cytotoxic Side Effects

**Figure 4:**
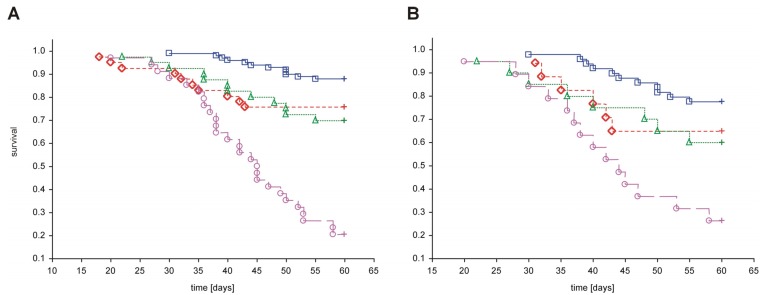
Kaplan-Meier curves of cumulative survival in animals treated intraperitoneally with PAMAM dendrimers G2, G3 and G4. The step function of the estimated cumulative fraction of surviving animals are given for pooled non-diabetic and streptozotocin-diabetic animals (**A**) or STZ-diabetic animals (**B**) treated with vehicle or with various PAMAM dendrimers. Complete observations are marked with squares, diamonds, triangles and circles for vehicle, PAMAM G2, G4 and G3, respectively; censorships are marked by “+”. The significance of differences between survival curves (log-rank Peto test) for the combined strata (non-diabetic, diabetic), was *p* < 0.0001; *p* < 0.0001 for the chi^2^ test for the trend: *veh* < G2 = G4 < G3.

Despite the fact that PAMAM G2, G3 and G4 possess hypoglycaemic activity, these compounds were found to have an overall negative effect on animal survival [5,6,21]. Although the mortality rate varied considerably in different studies [5,19], administration of PAMAM G2, G3 or G4 was associated with significantly reduced survival. A brief overview of the effects on animal mortality observed in all *in vivo* experiments to date involving the use of PAMAM G2, G3 and G4 is presented in Figure 4. As shown in the figure, treatment with dendrimers was associated with increased mortality in both healthy and diabetic rats compared to animals with vehicle. Exposure to PAMAM G3 appeared to be the most toxic, whereas PAMAM G4 and G2 had considerably less impact on overall survival rate.

**Figure 5:**
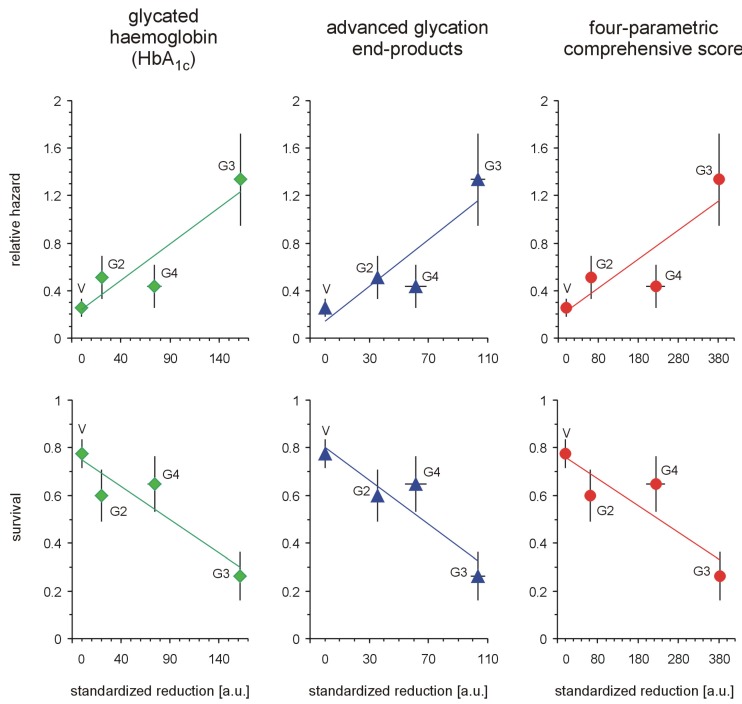
Relationships between standardised reductions in hallmarks of severe hyperglycaemia, relative hazard and overall survival in streptozotocin-diabetic rats treated with PAMAM dendrimers. The data, which are presented as the mean ± SEM (n = 5–19), represent the standardised reductions in hyperglycaemia/oxidation markers (per μmol NH_2_/kg body weight/day) in diabetic Sprague-Dawley rats treated intraperitoneally with PAMAM dendrimers. Kaplan-Meier survival estimates (survival rate and relative hazard) were estimated according to [22] ^1^ and [23] ^2^, respectively (for more details, see Figure 2 and the text). The associations (Pearson’s *r*_(P)_) between HbA_1c_, AGEs, CS and overall (cumulative) survival were, respectively: *r*_(P)__HbA1c_ = −0.661, *p* < 0.0001, *r*_(P)__AGEs_ = −0.527, *p* < 0.002, and *r*_(P)__CS_ = −0.550, *p* < 0.001; between HbA_1c_, AGEs, CS and cumulative relative hazard, *r*_(P)__HbA1c_ = 0.690, *p* < 0.0001, *r*_(P)__AGEs_ = 0.551, *p* < 0.001, and *r*_(P)__CS_ = 0.575, *p* < 0.0005.

^1^ Survival rate is expressed as the survivor function (S): S(t) = (number of individuals surviving longer than *t*)/(total number of individuals studied), where *t* is a time period known as the survival time, time to failure or time to event (such as death). Some authors present *S* as the estimated probability of surviving to time *t* for individuals alive just before *t* multiplied by the proportion of subjects surviving to *t*; ^2^ The cumulative hazard function (*H*) is the risk of event (e.g., death) at time *t*: *Ĥ*(*t*) = −ln(*Ŝ*(*t*)).

If we assume that toxicity increases in proportion to the polycationicity of the dendrimers, the observed toxic effects should increase in the order: PAMAM G4 (33 μmol NH_2_ groups/kg/day) < G3 (93 μmol NH_2_ groups/kg/day) < G2 (197 μmol NH_2_ groups/kg/day). Our findings, however, did not precisely match these expectations: although PAMAM G3 was indeed more toxic than PAMAM G4, the toxicity of PAMAM G2 did not exceed that of G3. Furthermore, the trend in dendrimer toxicity essentially parallels the ability of the various dendrimers to reduce the monitored hallmarks of severe hyperglycaemia, as shown in Figure 5 for three markers of hyperglycaemia: glycated haemoglobin, AGEs and the four-parametric comprehensive scores.

**Figure 6:**
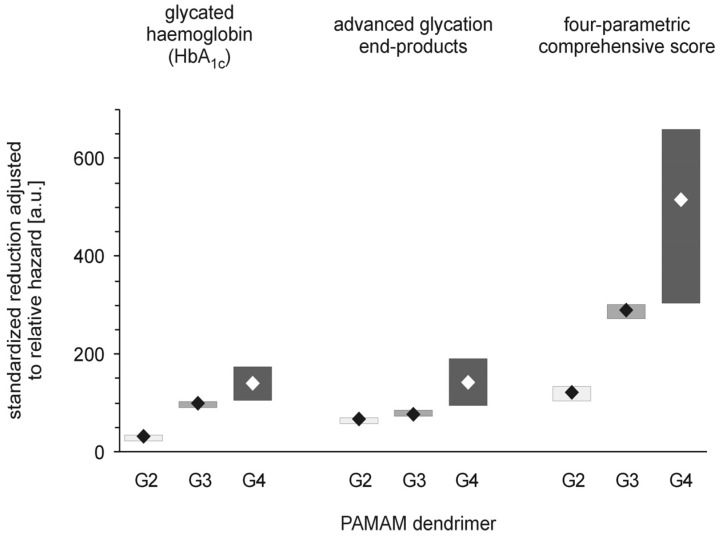
PAMAM dendrimer-induced reduction in markers of hyperglycaemia adjusted to overall mortality in streptozotocin-diabetic rats. The data, which are presented as median and interquartile range (lower 25% quartile to upper 75% quartile; n = 5–19), represent standardised reductions (per μmol NH_2_/kg body weight/day) in hyperglycaemia/oxidation markers expressed in arbitrary units, a.u., after adjustment for overall mortality (relative hazard) in diabetic Sprague-Dawley rats treated intraperitoneally with PAMAM dendrimers. Relative hazard was estimated using the Kaplan-Meier method (for more details, see the text and the legends to Figure 2 and Figure 5). The significance levels of the observed differences were: HbA_1c__adj_ _hazard_, *p* < 0.01, G2 *vs.* G3, *p* < 0.04, G3 *vs.* G4, *p* < 0.001, G2 *vs.* G4; AGEs _adj_ _hazard_, *p* < 0.02, G2 *vs.* G3, *p* < 0.0001, G3 *vs.* G4, *p* < 0.0001, G2 *vs.* G4; CS _adj_ _hazard_, *p* < 0.002, G2 *vs.* G3, *p* < 0.05, G3 *vs.* G4, *p* < 0.0001, G2 *vs.* G4.

The polycationicity of the PAMAM dendrimers used in the experiment increases in the order G2 (16) < G3 (32) < G4 (64), and the rank order of the equivalent concentrations of surface amino groups they provide at the doses used in the experiment is the reverse: 33 (G4) < 93 (G3) < 197 (G2) μmol/kg/day. Overlay of this tendency on the observed standardised reduction in the markers of severe hyperglycaemia showed that these trends do not match. PAMAM G3, which was the most effective, displayed the highest toxicity, while the least effective dendrimer appeared to be G2, the toxicity of which was roughly equal to that of G4. An interesting overall picture of the relationship between the effectiveness of PAMAMs as hypoglycaemising agents and their toxicity appears, however, when the observed PAMAM-mediated reductions in various markers of severe hyperglycaemia are normalised to mortality. Figure 6 clearly demonstrates that “effectiveness” adjusted in this way was significantly highest for PAMAM G4 and lowest for PAMAM G2. Overall, our studies demonstrate the amine-group concentration-dependent toxic effects of PAMAM dendrimers and show that there is a generation-dependent response of rats to treatment with dendrimers, with PAMAM G4 showing the best effectiveness/toxicity ratio and PAMAM G2 showing the worst.

Three possible reasons for the increased mortality of the animals given dendrimers are dendrimer dose, experiment duration and the route of dendrimer delivery. Review of the above-described outcome of our studies as well as available literature data clearly shows that the daily administration of PAMAMs for 60 days may simply be too long or too intensive not only for diabetic animals, but also, although to a lesser extent, for their healthy non-diabetic counterparts. Thus, while the significance of the impact of the doses used in our experiments might be minimised or even excluded, the experiment duration and the route of dendrimer delivery are far more likely to contribute to such the observed increases in animal mortality.

Although the characterisation of dendrimers in studies using cell cultures was a fundamental step forward, revealing several crucial features of these polymers, more specific preclinical applications require much more direct, reliable and representative models. Prior to 2008, the majority of reports on the effects of PAMAMs on animals were authored by Roberts at al., Neerman *et al.*, Malik *et al.*, Gupta *et al.*, and Okuda *et al.*, at that time, their reports were the only source of knowledge about the use of dendrimers in animals [16,17,18,24,25]. Based on the outcomes reported in these papers, it may generally be concluded that cationic PAMAM dendrimers of the lower full generations (G3 and G5) administered to C57 mice at doses of 2.6 mg/kg and 10 mg/kg, respectively, once a week for up to 6 months, demonstrated no negative effects, including behavioural changes or weight loss, and all the tested animals survived the experiment. However, when the dendrimers of a higher generation (PAMAM G7) were used at a dose of 45 mg/kg in the same time regime, the overall survival of the animals was reduced by 20% [18].

It has been reported that cationic dendrimers circulate in the blood for a much shorter time than their anionic counterparts; the authors of that study claimed that this could be the most likely reason for their limited toxic effects compared to other types of dendrimers [26]. The effects of the dendrimer based on melamine, given either as a single dose of 160 mg/kg or three intraperitoneally administered doses every 3 weeks over a period of 6 weeks, on renal and hepatic function were monitored in C57 mice by Neerman *et al.* The acute high-dose study was fatal in 100% of cases within 6–12 h after injection; however, neither acute nor subchronic administration resulted in significant renal damage. Although hepatic function remained unaltered at doses of up to 10 mg/kg dendrimer, extensive liver necrosis and significant increases in alanine aminotransferase activity occurred at a dose of 40 mg/kg [17]. Taking all of the above findings into account, it appears ambiguous and rather unlikely that the reduced survival rates observed in our studies are primarily due to toxic effects of PAMAM dendrimers in Wistar and Sprague-Dawley rats.

In summary, in contrast to our expectations, overall animal survival was greatly reduced after PAMAM dendrimer treatment, despite the fact that these agents demonstrated distinct protective effects and several markers of glucose toxicity in diabetes were vastly decreased [5]. In later sections of this review, we present evidence concerning the possible reasons for the surprising divergence between these two PAMAM-mediated effects.

#### 2.1.2. Overall Toxicity in Dendrimer Administration—An Effect of Specific Route of Dendrimer Delivery

It now appears that the most ambitious challenges with respect to the effective *in vivo* use of dendrimers are based on adjusting dendrimer generation, dose and treatment duration in such a way as to avoid the detrimental impact of dendrimers on a whole organism or on specific organs.

Because there is currently great commercial interest in the use of dendrimers in various pharmaceutical products, the evaluation of their toxicity towards mammalian cells in the course of both occupational or consumer exposure seems paramount at present. In our studies, as briefly described in earlier sections, we revealed the “double face” of the tested PAMAM dendrimers. In general, every experiment terminated with a significant reduction in the survival of animals to which dendrimers were administered, further reinforcing the belief that PAMAM dendrimers are most likely too toxic to be reasonably introduced into medical practice. Such uncertainty concerning the successful use of dendrimers contradicts the opinion commonly expressed in the literature, which promotes a wide range of applications for these polymers. However, a careful review of our results indicates another possible mechanism for increased PAMAM toxicity. It may be that dendrimers are not so toxic *per se* at the used doses; however, the route of dendrimer delivery may be a factor in determining animal mortality.

**Figure 7:**
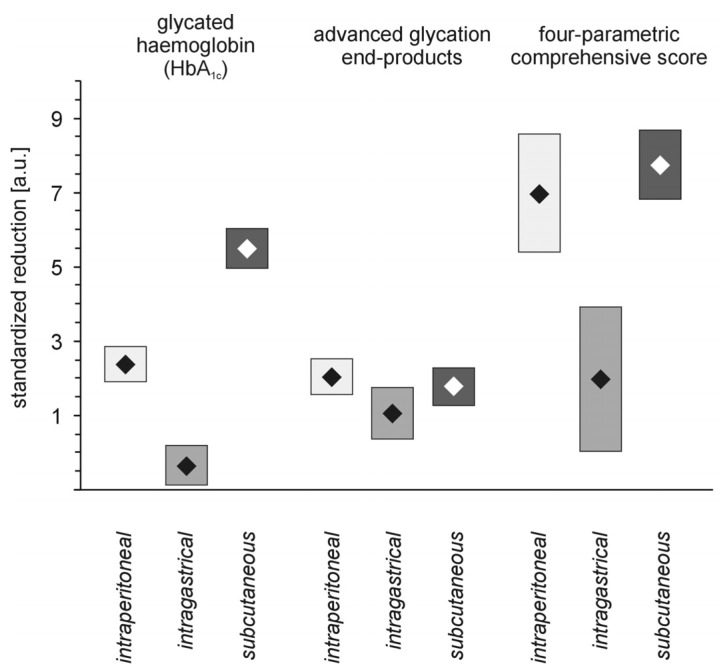
PAMAM G4 dendrimer-induced reductions in markers of hyperglycaemia in streptozotocin-diabetic rats treated with PAMAM G4 using various routes of delivery. The data, which are presented as the mean ± 95% CI (n = 5–19), represent standardised reductions in hyperglycaemia/oxidation markers in animals treated with PAMAM G4 dendrimers intraperitoneally, intragastrically or subcutaneously. The significances of the differences were: HbA_1c_, *p* < 0.0001, *Me*_subcutaneous_ > *Me*_intraperitoneal_ > *Me*_intragastric_; AGEs, *Me*_subcutaneous_ = *Me*_intraperitoneal_ > *Me*_intragastric_; CS, *p* < 0.0001, *Me*_subcutaneous_ = *Me*_intraperitoneal_ > *Me*_intragastric_.

**Figure 8:**
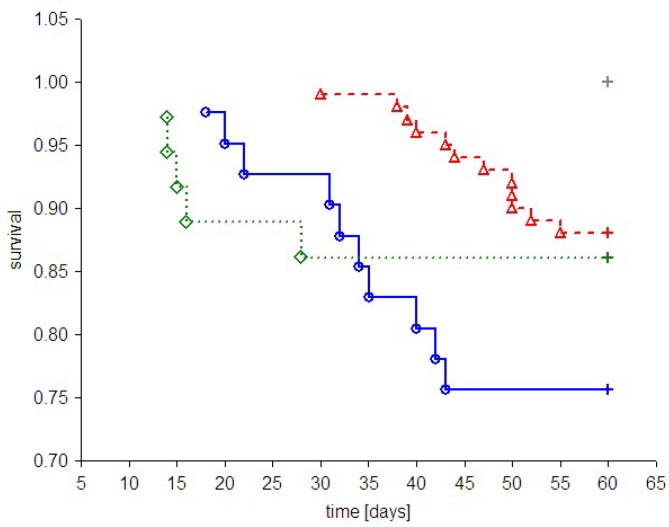
Kaplan-Meier curves showing cumulative survival in animals that received vehicle or PAMAM dendrimer G4 by various routes of delivery. The step function of the estimated cumulative proportions of the surviving rats are given for pooled non-diabetic and streptozotocin-diabetic animals treated with vehicle or PAMAM G4 using three different routes of delivery. Complete observations are marked with circles, triangles and diamonds for PAMAM G4 given intraperitoneally, vehicle given intraperitoneally and PAMAM G4 given intragastrically, respectively; censorships are marked by “+”. For the remaining conditions (vehicle or PAMAM G4 given subcutaneously, vehicle given intragastrically), no death events were recorded. The significance of the differences between survival curves given by the log-rank (Peto) test for the combined strata (non-diabetic, diabetic) was *p* < 0.01; *p* < 0.0001 for the chi^2^ test for the trend: G4 _intraperitoneal_ > G4 _intragastric_ > vehicle _intraperitoneal_.

The perception of PAMAM dendrimer activity in laboratory animals has “revolutionised” our way of thinking and influenced the design of subsequent experiments that tested three different routes of dendrimer administration to animals with experimental diabetes. What was the rationale behind the experiment? It has been shown that both anatomy and physiology present barriers to the accessibility of molecules to the target site and that the effectiveness of these barriers depends on the route of administration. In this regard, the same dendrimers administered by different routes could show differentiated biodistributions and pharmacokinetic profiles, thus resulting in altered therapeutic efficacy and side effects [27]. In our investigation, the most effective routes of administration for suppression of long-term markers of hyperglycaemia were intraperitoneal or subcutaneous; the intragastric route appeared to be the least effective (Figure 7). Nevertheless, the mortality of animals treated with dendrimer was highest in the groups of rats that received either a vehicle or PAMAM G4 intraperitoneally, regardless of whether the rats were non-diabetic or streptozotocin-diabetic rats (Figure 8).

In contrast to intraperitoneal administration of PAMAM G4, the subcutaneous method appeared to be the safest route for dendrimer delivery: no fatal events were recorded in this group of animals. Intragastric delivery of PAMAM G4 resulted in a moderate fatality rate, however; in addition, it was completely ineffective in reducing the markers of severe hyperglycaemia. In contrast, the “hypoglycaemising” effectiveness noted in animals given intraperitoneal or subcutaneous injections were almost equally high (Figure 7).

**Figure 9:**
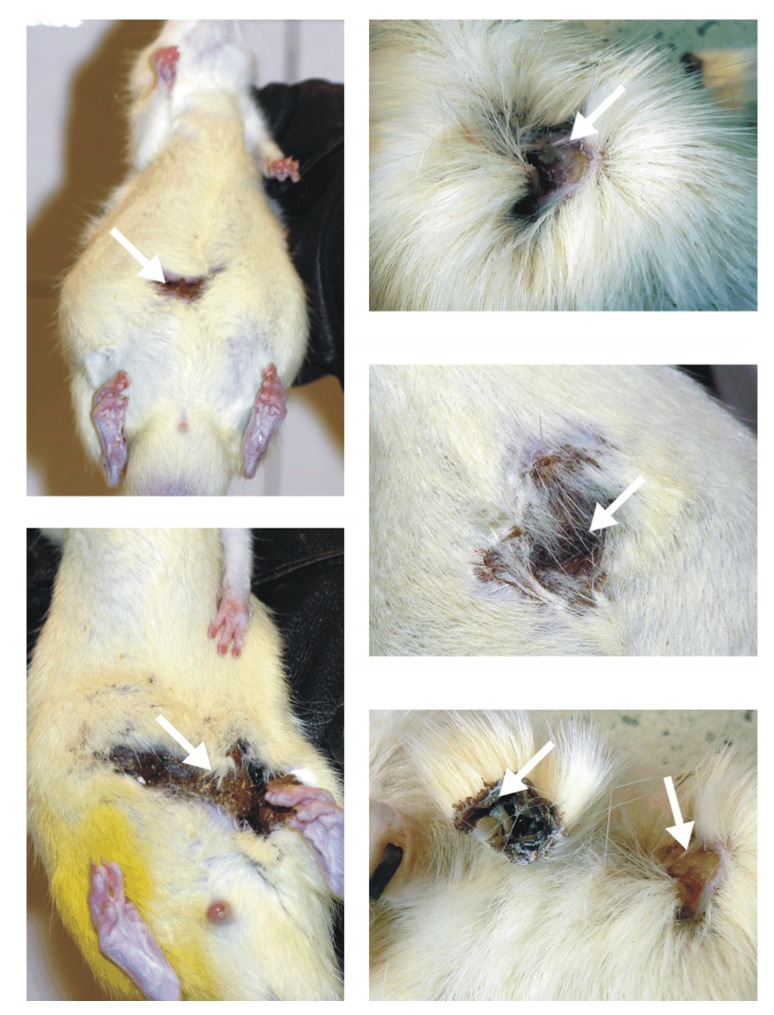
Skin irritation and inflammation in animals given PAMAM G4 dendrimers by intraperitoneal injection. The arrows indicate hair loss, reddening of the skin and abscesses filled with sanguine pus. For a description of the experimental design, please see the text.

While the intragastric method of treatment with PAMAM G4 resulted in the overall survival of 86.1 ± 5.8% of the animals (compared to 100% for vehicle), overall survival for the intraperitoneal route of delivery was significantly lower (75.6 ± 6.7%, compared to 97.2 ± 2.7% for vehicle) (*p* < 0.05 by log-rank test for the stratum of diabetic animals). These outcomes clearly suggest that the toxicity of PAMAM G4 dendrimers in rats largely depends on the delivery route chosen. Furthermore, our findings demonstrate that of the three tested routes of delivery, intraperitoneal delivery appears to be the most harmful. Another convincing piece of evidence supporting this reasoning is presented in Figure 9. It was shown that rats injected daily with the tested chemicals often experienced painful skin irritation and/or inflammation that was associated with hair loss, reddening of the skin, festering and open wounds and abscesses that were often filled with blood and pus. These skin changes occurred not only in diabetic rats but also in healthy animals; furthermore, they occurred not only after dendrimer treatment, but also in animals given vehicle.

Interestingly, the *in vivo* toxicity of PAMAM dendrimers, which has been extensively described in the literature, is commonly linked to the toxicity of dendrimer terminal groups, *i.e.*, toxicity is thought to depend on the dendrimer generation or on the chosen dose, but rarely on the manner of delivery [28]. The majority of studies indicate that dendrimer toxicity is dose- and generation-dependent, with higher doses and higher generations of dendrimers producing more profound toxicity *in vivo*. Additionally, the authors of this review agree that the *in vivo* toxicity profiles of dendrimers are closely related to the chemical structures of the dendrimers, their size and generation, the duration of exposure, the biodistribution of the compounds and the rate, location, and mechanisms by which they are metabolised. Although various routes of administration of dendrimers have been investigated, to the best of our knowledge, no studies have compared different routes of dendrimer delivery using the same animal model, the same dendrimer dose and similar environmental/laboratory conditions. Moreover, most studies carried out to evaluate the *in vivo* toxicity profile of dendrimers have focused on the overall survival rates of mice or rats and changes in their body weight, level of appetite and other behavioural factors. In contrast, our studies were aimed not only at monitoring animal survival after exposure to dendrimers, but also at selecting the most effective and the least harmful method of dendrimer administration. In the course of our studies on full-generation PAMAM dendrimers, the “Janus face” of these agents was revealed: on one hand, animals given dendrimers exhibit reduced survival rates, while on the other hand PAMAMs have the ability to scavenge excess blood glucose and to reduce the hallmarks of severe hyperglycaemia. Our results show that intraperitoneal administration of dendrimers contributes to much higher animal mortality, although all of the parameters reflecting severe hyperglycaemia improved considerably in the animals that survived the experiment. On the other hand, the intragastric route of dendrimer administration was the least efficient in reducing the markers of diabetes-associated metabolic impairments, although it was also relatively non-toxic.

A number of research groups have shed light on the potential toxic effects associated with the administration of PAMAM dendrimers *in vivo* by repeated injection during long-lasting treatments [22,29,30,31]; the results of these groups’ studies indicated that oral administration might be safer [32]. Based on our experience with dendrimers under *in vivo* conditions, we essentially agree with these suggestions. Nevertheless, we wish to note that administration of dendrimers by a route that renders them devoid of their curative impact greatly limits their clinical usefulness in medicine. Very few reports are available on the ability of dendrimers to cross gastrointestinal membranes. Wiwattanapatapee *et al.* investigated the transport of PAMAMs across the intestinal wall in adult rats using ^125^I-labeled dendrimers. The results of that study suggested that dendrimer transport across the intestinal membrane is charge-dependent. In general, for cationic dendrimers, it has been reported that tissue uptake is higher than serosal transport. However, the tissue uptake of anionic dendrimers was considerably higher than that of cationic ones [33]. The conclusions drawn by other authors [34,35] strongly support the use of the oral route of dendrimer delivery, and the accumulated data show that PAMAM dendrimers are able to cross gastrointestinal membranes; thus, they may be useful in various medical treatments. From our point of view, oral dendrimer supplementation did not prove useful in the model of experimental diabetes mellitus. Indeed, although intragastric administration did not result in greatly reduced animal survival, dendrimers administered in this way did not appear to efficiently scavenge excess glucose. However, we are aware that our present reasoning is based on findings derived from a single experiment involving intragastric delivery of the dendrimer. Hence, more detailed studies comparing various previously studied routes of dendrimer delivery are definitely required, as are studies on other routes of delivery that have not yet been explored.

Lastly, the subcutaneous administration of PAMAM dendrimers has proven to be the best compromise between toxicity and the anti-glycation potential of PAMAM dendrimers. Overall, it seems that when considering the use of PAMAM dendrimers as pro-pharmaceutics in the treatment of hyperglycaemia in diabetes, we need not be forced to choose “the lesser of two evils”. The subcutaneous route of administration definitely appears to be the optimal choice, at least for PAMAM G4. The question of whether this rationale is also valid for other generations of PAMAM dendrimers is open for future investigation. Nevertheless, the existing data emphasise that the medical potential of dendrimers is most fully realised when they are administered *via* a route that maintains their marked effectiveness and produces low toxicity. Furthermore, the toxicological status of dendrimers should be more conclusively established before any final conclusions are drawn in this regard.

#### 2.1.3. Mitochondria under the Direct and Indirect Fire of PAMAM Dendrimers

Mitochondria are well-known for their pivotal role in cellular metabolism associated with the production of ATP; however, mitochondria also play a key role in the maintenance of the fragile balance between cell survival and death. Therefore, it seems desirable to routinely make sure that potential therapeutic agents do not induce any undesired toxic effects associated with drug-mitochondria interactions, particularly in the context of the observed dendrimer-mediated reductions in the hallmarks associated with severe diabetic hyperglycaemia.

Our first predictions regarding the impact of PAMAMs on mitochondria were based on a study by Malik *et al*. in which the toxic nature of amino-terminated PAMAM dendrimers was revealed. Using the MTT assay, which monitors the mitochondrial oxidoreductase activity, the authors demonstrated that PAMAM dendrimers (G1-G4) exhibited a generation-dependent cell toxicity [16]. Nevertheless, for rather a long time thereafter, no other reports concerning the impact of dendrimers on various aspects of mitochondrial activity were published. Now, additional experimental data generated by a number of independent research groups, including our team, have become available [4,6,21,36,37,38,39,40]; these data show that PAMAM dendrimers may have considerable impact on the function of mitochondria.

**Figure 10:**
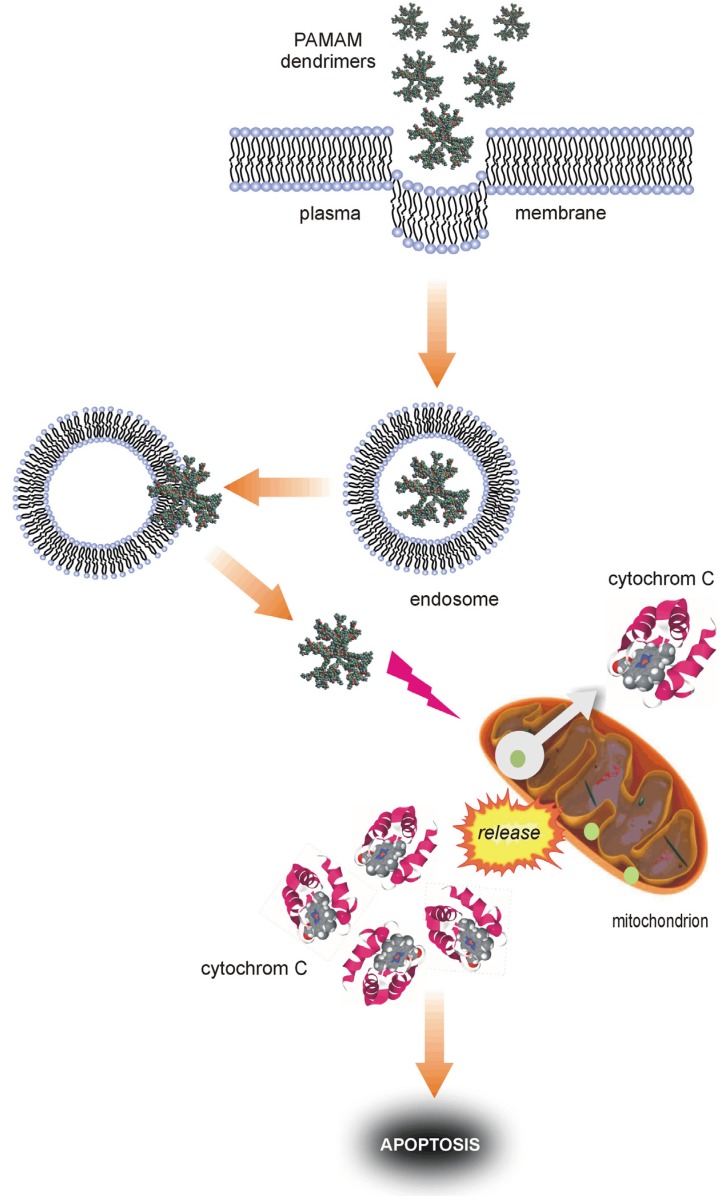
Possible mechanisms of the pro-apoptotic activity of PAMAM dendrimers.

Some reports have provided fairly convincing evidence that treatment with PAMAM dendrimers can lead to apoptotic cell death [36,37,39,41]. Lee *et al.* showed that poly(amido)amine dendrimers of the 4th generation penetrated membranes of the human lung cell line WI-26 VA4, dispersed in the cytoplasm and then entered the mitochondria, where they subsequently contributed to the release of cytochrome c, increased Bax expression, decreased Bcl-2 expression, reduced mitochondrial membrane potential and activated pro-apoptotic caspases. An obvious consequence of these events was an observed increase in the number of apoptotic cells [36]. Similar observations involving the use of 5th-generation PAMAM dendrimers in cervical carcinoma (HeLa) cells were reported by Thomas *et al.* These authors showed that PAMAM dendrimers induced changes in mitochondrial membrane potential and led to caspase activation and cell apoptosis [39]. Nyitrai and co-workers confirmed that 5th-generation PAMAM dendrimers have adverse effects on mitochondria. Exposure of hippocampal astroglial cells to PAMAM resulted in intracellular calcium mobilisation and mitochondrial membrane depolarisation [42]. Mitochondrial toxicity upon treatment with PAMAM dendrimers (G4-G6) was also noted and reported by other researchers using various mammalian cell lines [38,41]. The results showed that cationic PAMAM dendrimers became localised in mitochondria, where they stimulated a mitochondria-dependent cytotoxic response involving significantly increased ROS generation and apoptotic cell death in a generation-dependent manner. The results of a recent study by Mukherjee *et al.* in a human keratinocyte line (HaCaT) largely explain some molecular aspects of PAMAM dendrimer cytotoxicity. According to the authors, the interaction of cells with PAMAM dendrimers likely occurs through a two-step process. The dendrimers first become entrapped in endosome-like structures; they are then released in the proximity of mitochondria. After the relocation of the dendrimers to mitochondria, there is concomitant overproduction of ROS, release of cytochrome c and caspase activation, and apoptosis may occur [37]. A diagram representing such a process is shown in Figure 10.

Our own research also did not show PAMAM dendrimers in the best light. To determine the effects of dendrimers on rat heart and liver mitochondria impaired by long-lasting experimental diabetes, we monitored several parameters that reflect the bioenergetics of mitochondria, including RCR, OPR, P/E, L/E and ADP/O (assessed in a standardised experimental regime). The *in vitro* studies, which were based on the measurement of oxygen consumption by isolated mitochondria treated with PAMAMs (G2, G4) under conditions involving various inhibitor-modulated functional states (blocked complex I, inhibited ATPase, *etc.*) revealed significant changes in selected mitochondrial parameters such as RCR, an index of coupling and an indicator of mitochondrial membrane functional integrity that is useful for the detection of mitochondrial defects, OPR, the oxidative phosphorylation rate, and ADP/O, the coefficient of oxidative phosphorylation. *In vitro* incubation of isolated rat liver mitochondria with PAMAM G2 and G4 caused a deterioration in mitochondrial function that was reflected in a reduced RCR ratio. In turn, the observed decreased OPR and ADP/O rates suggest that incubation of mitochondria with dendrimers also impaired the process of oxidative phosphorylation, thus limiting ATP production [6]. The results also showed that mitochondria are more sensitive to PAMAM G4 than to PAMAM G2 dendrimers [4].

In another study aimed at demonstrating the impact of 3rd-generation PAMAMs on the bioenergetics of rat cardiomyocyte mitochondria, no direct harmful *in vitro* effect of 100 µmol/L PAMAM G3 on the heart mitochondrial RCR ratio was found. However, the increased P/E ratio together with the decreased electron transport system (ETS) capacity in the treated mitochondria indicated that oxidative phosphorylation was impaired, although the amount of ATP formed per atom of oxygen consumed (ADP/O) remained unchanged. Moreover, exposure of mitochondria to PAMAM G3 dendrimers led to increased mitochondrial uncoupling as evidenced by an elevation in the L/E ratio [21]. The alleviating effects of PAMAM G3 dendrimers were nicely demonstrated in *in vitro* experiments, in which the dendrimers were shown to improve the respiration of rat heart mitochondria incubated with the “carbonyl stress” agent—methylglyoxal (MGO) [21]. Importantly, the effects of PAMAM G3 on cardiomyocyte mitochondria varied significantly depending on the season (autumn/winter *vs.* spring/summer) in which the animals were euthanised. Interestingly, an impact of seasonality on mitochondrial function was also observed in other studies we conducted on experimental diabetes in rats over several seasons during a period of 4–5 years. The effect is most likely caused by the different sensitivities of mitochondria obtained in various seasons [43,44]. In *in vitro* studies aimed at evaluating the potency of various generations of PAMAM dendrimers (G2, G3 and G4) in attenuating the detrimental effect of experimental diabetes on rat heart mitochondrial respiratory capacity, the outcomes differed depending on the dendrimer generation and the tissue. PAMAM G4 (ca. 7.3 mg daily/kg b.w. for 60 days) significantly inhibited respiratory function in the ADP-stimulated state and lowered the OPR rate, indicating that it may reduce the efficiency of oxidative phosphorylation in heart mitochondria. However, PAMAM G4 did not affect the functional integrity of the mitochondrial membrane (RCR) or ADP/O (the amount of ADP phosphorylated to ATP per oxygen atom consumed by mitochondria). In contrast, liver mitochondria were shown to be resistant to the suppression of mitochondrial metabolism and phosphorylation by PAMAM G4 dendrimer, and their respiration was unchanged [6].

**Figure 11:**
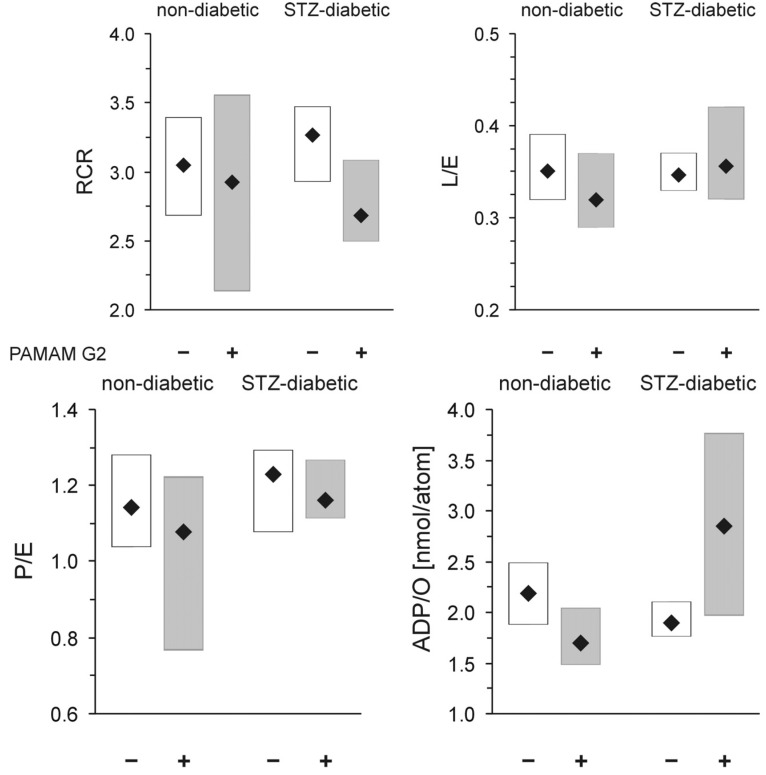
*In vivo* evaluation of the impact of PAMAM G2 on rat heart mitochondrial respiration. The data are expressed as median (diamonds) and lower-upper quartile range (rectangles); n = 8–20 animals. PAMAM G2 dendrimers (40 mg/kg/day) were administered to non-diabetic and streptozotocin-diabetic Sprague-Dawley rats for 60 consecutive days. The significance of differences was estimated using the non-parametric Kruskal-Wallis test followed by the Conover-Inman all-pairwise-comparisons test: * *p* < 0.03 “diabetic rats + vehicle” *vs.* “diabetic rats + PAMAM G2”; ** *p* < 0.0001 “healthy rats + PAMAM G2” *vs.* “diabetic rats + PAMAM G2”. *Abbreviations*: RCR, respiratory control ratio; L/E, leak control ratio; P/E, phosphorylation system control ratio; ADP/O, the amount of ADP phosphorylated to ATP per oxygen atom consumed by mitochondria in nmol ADP/nAtm O.

Mitochondria isolated from animals treated with PAMAM G2 (40 mg daily/kg b.w. for 60 consecutive days) were not significantly affected, regardless of the tested group (healthy or diabetic). As shown in Figure 11, the dendrimer significantly affected neither RCR, the index of mitochondrial coupling, nor L/E, the index of mitochondrial uncoupling), suggesting that PAMAM G2 lacks any substantial negative impact on the overall bioenergetic properties of mitochondria. The only parameter that changed significantly upon G2 administration was the formation of ATP (ADP/O parameter). We observed that upon treatment with the dendrimer, diabetic mitochondria produced more ATP than their healthy counterparts and more than diabetic subjects that received vehicle alone. The increased rate of ATP production observed upon exposure to PAMAM G2 could indicate an improvement in mitochondrial activity. On the other hand, however, this phenomenon, which was observed only in mitochondria from animals that had received the dendrimer, may simply mean that “overproduction” of ATP by heart mitochondria isolated from animals supplemented with PAMAM G2 is a sign of the toxic (negative) face of the dendrimer rather than of a health-promoting impact of the dendrimer on mitochondrial electron transfer activity. It should be mentioned here that quite recently an interesting view was offered concerning the rate of oxygen uptake and higher ATP production in response to treatment with various compounds. Although the observed increase in the energy state might be initiated by respiratory chain failure, the lack of changes in other parameters that were tested in parallel makes such reasoning questionable. ATP is a product of respiratory chain activity; therefore, when the ETS (electron transport system) is stimulated by externally added compounds (*i.e.*, drugs, chemicals), increased transmembrane potential, facilitated electron flow and ROS production may also be noted. A resulting finding might be a rise in ATP production due to moderate treatment, although we are, of course, aware of the fact that long-term exposure to such compounds is undoubtedly detrimental to mitochondrial function [45]. If mitochondria are not able to adapt rapidly enough to such conditions, reduced mitochondrial potential and decreased respiratory capacity may occur. Overall, long-lasting treatment with dendrimer G2 can provoke a significant increase in ATP production, although other bioenergetic parameters may not change [46]. Nevertheless, our findings did not indicate that PAMAM G2 improves mitochondrial activity or that it normalises mitochondrial respiration that has been impaired by hyperglycaemia. It is highly probable that the higher ATP production observed upon prolonged exposure to the dendrimer is instead a result of a slight mitochondrial dysfunction, which may have just begun and which may worsen with time, finally leading to mitochondrial damage. Another possibility is that the mitochondria may adapt to dendrimer activity and make a “full recovery”.

Thus, our observations originating from *in vivo* studies on the effects of PAMAM G4 and PAMAM G2 in experimentally diabetic rats diverge in that they show every pronounced impairment in mitochondrial function for G4 and, paradoxically, beneficial shifts in mitochondrial function in the case of G2. Therefore, we found it very tempting to investigate the effects of PAMAM G3, which is located in the middle with respect to polycationicity. The effect of administration of dendrimer G3 (20 mg/kg/day for 60 consecutive days) on the activity of heart mitochondria was examined in another set of experiments similar to those involving G2 and G4 [21]. Regrettably, most of the dendrimer-treated animals died before the termination of the experiment; the high toxicity of PAMAM dendrimer G3 at the applied dose vastly reduced the size of the examined sample and made it impossible to draw any reliable conclusions from these experiments.

**Figure 12:**
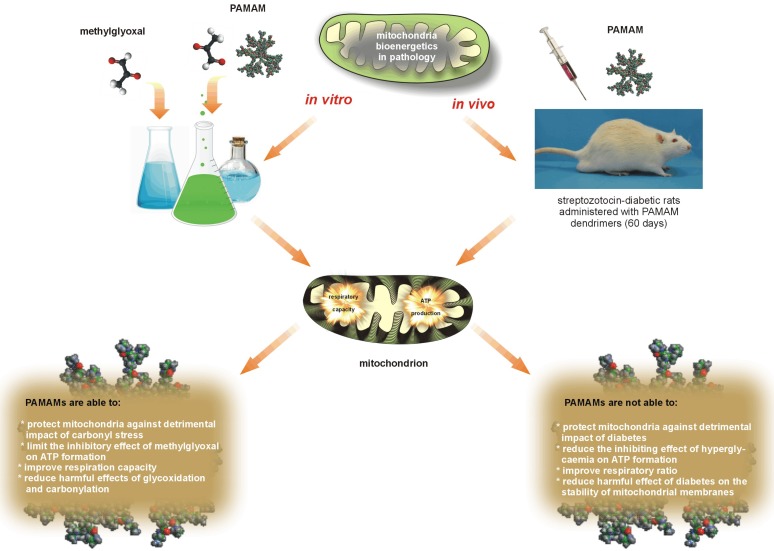
The two faces of PAMAM dendrimers used in restoring the impaired mitochondrial function observed under *in vitro* and *in vivo* conditions.

Careful review of data collected from *in vitro* [6,21,40,44] and *in vivo* studies [7,21] leads us to deduce that the overall effects of dendrimers depend most strongly on the dendrimer concentration (dose) and the duration of exposure used. Although most of our outcomes showed that PAMAM dendrimers had a negative impact on mitochondrial function, the suggestion was raised that under well-designed experimental conditions PAMAMs might be successfully used in the limitation of mitochondrial disorders caused by glycoxidative and carbonyl stress [21] or by overload with calcium ions [40,44]. A brief collation of *in vitro* and *in vivo* assessments of dendrimer activities in mitochondria is schematically presented in Figure 12.

In summary, the literature data briefly discussed above clearly demonstrate the toxic effects of full-generation PAMAM dendrimers on mitochondrial function. These adverse effects are likely to be related to the chemistry of these nanoparticles and, more specifically, to the presence of a large number of amino groups. The cationic charge of the surfaces of PAMAM dendrimers may promote their interaction with mitochondrial membranes [47,48] that consist of a lipid bilayer and membrane proteins [49,50], leading to hole formation, rearrangements of mitochondrial permeability transition pores and disruptions in the mitochondrial electron transport chain. Irrespective of the detailed mechanisms of these changes, full-generation PAMAM dendrimers have shown their “grim face” as compounds that strongly interfere with proper mitochondrial function. Because the majority of life processes depend on mitochondrial functionality, we have to argue that the overall picture of the interactions of PAMAM dendrimers with mitochondria concurs with the general message of this review, which is that the potential use of these compounds in pharmacology may be limited.

### 2.2. Use of PAMAM Dendrimers to Reduce Complications Associated with Nonenzymatic Modifications of Biomacromolecules—A Focus on Alzheimer’s Disease

#### 2.2.1. Evidence for the Coincidence of Alzheimer’s Disease and Type 2 Diabetes Mellitus

Alzheimer’s disease (AD), the most common human amyloidosis, is characterised by depositions of intracellular neurofibrillary tangles and extracellular fibrillar amyloid proteins that result in neuronal loss and in the development of serious neuropsychological deficits. Few hypotheses regarding the pathogenesis of AD have been proposed [51]. Importantly, the significance of nonenzymatic modifications of biomacromolecules in AD has been recently noted, and such modifications are increasingly regarded as a possible common denominator in the pathogenesis of AD and of type 2 diabetes mellitus (T2DM).

The concept of a putative molecular overlap between AD and T2DM derives from a study that revealed a higher prevalence of impaired glucose tolerance and diabetes among AD patients [52]. This observation suggests that AD patients are more prone to experience impaired glucose metabolism; one possible explanation for this phenomenon could be the reduced intracerebral insulin level and impaired insulin signalling that is observed in AD patients [52,53,54]. The considerable overlap of the impairments of insulin signalling reported in type 1 and type 2 diabetic patients and AD patients has even led some researchers to refer to AD as “type 3 diabetes mellitus” [52,54,55].

One of the most intriguing features of the association between diabetes and AD is not only the observation that AD subjects demonstrate a higher incidence of diabetes but also the fact that poorly controlled diabetic patients that are non-demented may become more vulnerable to neurodegeneration and to cognitive impairment with time. Chronic exposure to excessive glucose results in increased risk of AD [56] and decreases the time period over which transition from mild cognitive impairment to severe dementia occurs. The epidemiological data, which were further confirmed by the use of histological and neuroimaging techniques [57,58,59], have clearly demonstrated that the risk of mental illness is significantly increased in poorly controlled diabetes [60] and that preexisting diabetes is associated with earlier development of the onset of dementia and decreased survival time following the beginning of dementia [61].

A common feature of AD and diabetes is the occurrence of advanced glycation end products (AGEs), which may be effectively formed in the brain under conditions of hyperglycaemia; myelin [62], neurons [63,64] and glia [65] are particularly susceptible to easily form AGEs in certain brain regions such as the hippocampus and cerebral cortex [63,64]. Interestingly, the intracerebral AGEs detected in AD subjects do not represent a homogenous group of compounds. In addition to β-amyloid, also tau protein and apolipoprotein E may undergo non-enzymatic glycosylation, as has been detected in AD plaques [66,67]. The implications of AGE formation in both comorbidities (diabetes and AD) due to glucose metabolism disorders (AD) and higher incidence of mental disturbances under conditions of prolonged hyperglycaemia (diabetes) are reinforced by the fact that treatment of hypoglycaemia not only mitigates the metabolic impairments of diabetes but also reduces the severity of memory, cognition and behaviour impairments in these patients. This consequently leads to the quite surprising conclusion that AD progression may be less severe when the disease coincides with T2DM. The unexpected “paradoxical” observation that T2DM alleviates AD symptoms can be explained by the use of insulin by diabetic patients and by the use of other drugs that sensitise brain cells to insulin action [68]. Thus, insulin appears as one of the central molecules in anti-AD treatment. Insulin also interact with other anti-diabetic drugs such as metformin, which, without insulin, may even exert neurotoxic rather than neuroprotective effects [69].

#### 2.2.2. β-Amyloid and Advanced Glycation end Products in “the Enchanted Circle” of Interdependencies

β-Amyloid seems to remain upstream in the process of AGE formation in the central nervous system, whereas albumin-derived AGEs may be synthesised in activated microglia after exposure to β-amyloid [70]. As in the central nervous system, in peripheral tissues β-amyloid can regulate the production of AGEs, as it does in the central nervous system. Thus, glucose metabolism becomes impaired in ongoing AD not only due to the dysfunction of central insulin-dependent pathways in the hypothalamus but also as a result of direct interactions of β-amyloid with peripheral tissues that are pivotal for glucose metabolism. These interactions are most likely mediated not by brain-derived β-amyloid, but by a pool of this mediator that is present in peripheral tissues and that apparently increases after glucose loading [65]. Such glucose loading facilitates the formation of AGEs, potent factors in inducing the generation of reactive oxygen species *via* the NADPH oxidase-dependent pathway [71]. ROS further mediate the synthesis of the amyloid precursor protein (APP) [72], which, in turn, is processed to the β-amyloid protein end product. β-amyloid, the synthesis of which is stimulated by AGEs, can react with a plethora of glycosylating agents that increase the rate of its polymerisation and self-aggregation [73,74]. Importantly, when β-amyloid is present in the AGE-enriched extracellular matrix, its interaction with cell membranes becomes robustly facilitated [75].

Thus, β-amyloid can be considered an upstream molecule that directs the synthesis of AGEs. In addition, AGEs have also been characterised as amyloidogenicity “supervisors”. Which compound, β-amyloid or AGEs, is the more potent “second generation” regulator is not known. Interestingly, however, both of these molecules are potentially targeted by PAMAM dendrimers.

#### 2.2.3. Can PAMAM Dendrimers Break “the Enchanted Circle” of Interactions between β-Amyloid and Advanced Glycation End Products?

Undoubtedly, dendrimeric nanocompounds can play a dual role: they may act as disruptors of amyloidogenicity, as has been shown in some very simple *in vitro* systems; they may also “check out” as effective scavengers of AGEs, as has been shown in both *in vitro* and *in vivo* models. In disrupting the formation of amyloid structures, PAMAM dendrimers appear to act as thermodynamic inhibitors. This activity of PAMAM dendrimers results in much reduced final concentrations of β-amyloid fibrils; however, the dendrimers have no impact on the rate of fibril formation. Changes in the concentrations of β-amyloid fibrils may be attributed to the unspecific binding of PAMAM dendrimers to the free termini of protein fibrils, thus leading to “scavenging” of peptide monomers and prevention of their crosslinking. Moreover, PAMAMs may disrupt β-amyloid aggregates that have already been formed, thus reinforcing their prevention of fibril formation. It has occasionally been reported that PAMAM dendrimers affect the rate of nucleation or elongation of β-amyloid fibrils [76]. These preliminary *in vitro* observations reinforce the idea that PAMAM dendrimers may potentially represent both anti-amyloidogenic and anti-Alzheimer’s agents. The important question, which still remains unresolved, is whether these expected beneficial properties would occur in a living organism when PAMAM dendrimers are administered through the general circulation and not directly to the brain. Such a systemic action seems unlikely at first sight; however, some researchers have presented arguments against this idea. It has been argued that the distribution of PAMAM dendrimers to peripheral organs and tissues is unlikely, even more so considering the fast outflow of PAMAM dendrimers from the vascular bed, which apparently limits the chance of blood-brain barrier (BBB) passage, followed by the interactions of dendrimers with brain parenchyma*.* We do not have an attractive idea at present on how PAMAM dendrimers could possibly interact with extracellular amyloid-β deposits embedded in senile plaques in brain parenchyma. Specifically, the question of how these nanocompounds could possibly “find” their target amyloid proteins, leaving other proteins and molecules unaffected, remains a mystery. Such targeting seems rather unlikely or even impossible in the central nervous system environment, which is rich in charged molecules that can easily interact with highly polycationic PAMAM dendrimers. Hence, it is doubtful that the anti-amyloidogenic effects of PAMAMs could be achieved unless the surfaces of the dendrimers were modified to contain some directing residues. The presence of such a layer over the PAMAM surface, however, would also inevitably lead to the occupation of free primary amino groups, which are the most important and crucial chemical characteristics of PAMAMs and which are responsible for the interactions of dendrimers with amyloidogenic proteins. The importance of possible modifications of native dendrimer surfaces for the maintenance or loss of some crucial properties has been clearly illustrated by Kaminskas and co-workers, who observed a dramatically elevated accumulation of surface PEG-ylated polylysine dendrimers in lymph nodes [77] in contrast to non-PEG-ylated molecules.

**Figure 13:**
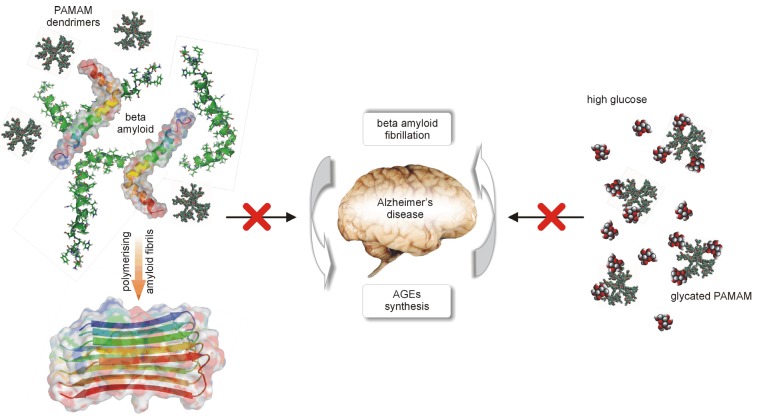
PAMAM dendrimers disrupt the association between the formation of advanced glycation end-products (AGEs) and β-amyloid fibrils. In parallel with reduced formation of fibrils of β-amyloid, diminished AGE synthesis occurs in the brain affected by Alzheimer’s disease. In addition, PAMAM-mediated scavenging of excess glucose in the peripheral nervous system decreases the vulnerability of neurons to amyloidosis.

In human subjects, β-amyloid can also be detected in blood plasma, where it may even serve as a predictor of AD development [78]. Peripheral β-amyloid is not an inert player in AD development. It can increase the permeability of BBB through the breakage of the zonula occludin-1 and the “opening” of intercellular fenestrae in the endothelial layer of the BBB, thus permitting uncontrolled leakage of molecules from the blood into the brain parenchyma. Interestingly, the process that alters brain homeostasis may be initiated by interactions between β-amyloid and AGE receptors (RAGEs) [79]. However, an intriguing issue remains unaddressed concerning the possibility of AD regression following the disruption of β-amyloid structures in peripheral tissues by PAMAM dendrimers. It is still unclear whether the half-life of PAMAM dendrimers in blood would be sufficient to achieve such an effect and whether the “fishing out” of dendrimers by other plasma proteins might considerably hamper this process (Figure 13). 

A much broader view of AD as “a disorder of protein folding” allows us to expand the “amyloid hypothesis” from the central nervous system to the pancreas, where β-cell apoptosis induced by the formation of amylin-derived amyloid has been reported [80]. Regrettably, the anti-amyloidogenic properties of PAMAM dendrimers in β-cells have not yet been investigated, even in *in vitro* systems.

In conclusion, PAMAM dendrimers may interfere with the formation of the inducers of two important pathways in AD pathogenesis. The first of these involves β-amyloid deposition in senile plaques and the disruption of β-amyloid deposits, the key morphological sign of this neurodegenerative disease, while the second involves β-amyloid-dependent AGEs formation. In the case of diabetes-related AD, such an approach may be considered a means of primary prevention in that scavenging of toxic glucose derivatives is expected to lead to decreased β-amyloid aggregation. In cases of clinically evidenced AD, such an approach might be regarded as a symptomatic treatment as a means of inducing the solubilisation of β-amyloid aggregates (a secondary prevention) as well as a compounding cure for the reduction of excessive blood glucose (as in a primary prophylaxis).

Whether the potential benefits associated with this aspect of PAMAMs are likely to outweigh their undesired toxic side effects remains a matter for further research and debate. It is important to consider the validity of using PAMAM dendrimers in anti-diabetes/anti-AD treatment, especially in the light of the aforementioned recently accumulated evidence indicating that insulin can be used as an anti-AD drug. The following question remains open: is justified to replace a natural hormone (or even its mimetic) that has been well characterised with respect to its risk/benefits ratio and has a well-described pharmacokinetic profile and known molecular mechanism with PAMAM dendrimers, which are agents with uncertain and ambiguous pharmacological characteristics?

#### 2.2.4. A Challenge for the Future: Possible Modulation of Blood-Brain Barrier Permeability by PAMAM Dendrimers as a Tool for the Prevention of Diabetes-Associated Alzheimer’s Disease?

**Figure 14:**
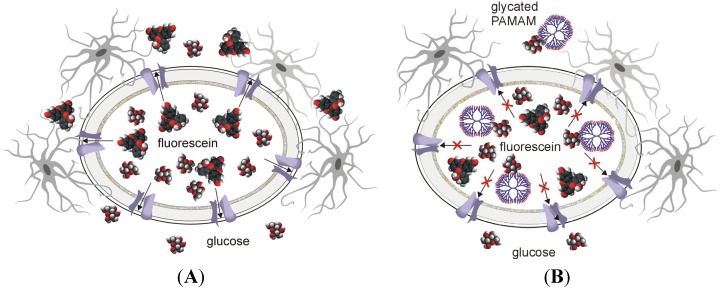
Ameliorating effects of PAMAM dendrimers on severe hyperglycaemia-mediated disruption of the blood-brain barrier. (**A**) Hyperglycaemia (glucose) disrupts blood–brain barrier (BBB) integrity, leading to leakage of low- molecular-weight compounds (fluorescein) from the blood into the brain parenchyma. (**B**) Dendrimers (PAMAM) facilitate the restoration of BBB integrity by scavenging excess glucose.

We have recently reported that PAMAM G4 dendrimers affect BBB permeability in streptozotocin diabetic rats. The modulation of BBB transporting features primarily concerned the movement of small solutes such as fluorescein (MW *ca*. 330 Da) or dextran-conjugated fluorescein (MW *ca*. 20 kDa) across the blood-brain barrier. This modulation clearly suggests that the BBB’s ‘structure’ (its intercellular connections) remains essentially undamaged in experimental diabetes, despite the fact that membrane endothelial transporters are affected (Figure 14).

Our experiments with α-cyano-4-hydroxycinnamic acid (CHCA), a selective inhibitor of type I monocarboxylate acid transporters, indicated that the transport of fluorescein might be mediated by MCT1; this result concurs with that of an earlier study by Berginc *et al.* [81].

These observations may point to higher MCT1 expression under conditions of long-term hyperglycaemia and correspondingly lower expression of these membrane carriers in the course of anti-diabetic treatment with PAMAM dendrimers. The final conclusion arising from our and other studies is that shifting between normo- and hyperglycaemia is probably associated with concomitant shifts in the expression patterns of energy substrate transporters: (a) monocarboxylate acids and MCT1, which dominate in hyperglycaemia, and (b) glucose transported by the glucose transporter 1 (GLUT1), which possibly plays a role after hypoglycaemising treatment. Such a suspicion is consistent with some earlier findings that clearly showed increased MCT1 expression in hyperglycaemia by the use of pharmacological and spectroscopic fluorescence approaches [82,83]. Additionally, a reduction in glucose concentration has been reported to increase GLUT1 expression in brain endothelial cells [84].

The changes observed after PAMAM dendrimer treatment are certainly secondary to the primary effects of these compounds on glycaemia control (fasting glucose levels and glycated haemoglobin concentration) [19] and PAMAM-induced modulation of glucose concentrations, which is likely to initiate either up- or down-regulation of energy substrate transporters, as observed in the model systems mentioned above.

These results may imply the existence of mechanisms that allow the balance between energy substrates to be maintained to achieve suitable levels of ATP synthesis in neurons. These cells are typically “glucose-addicted”; however, under some circumstances they prefer to use a particular monocarboxylate, lactate, as a fuel. Thus, it appears that the energy homeostasis in brain mitochondria largely determines the resistance to neurodegeneration. For that reason, maintenance of a metabolic balance between the supply of energy substrates and neuronal energy demands in hyperglycaemia is one of the crucial events in diabetes-related AD pathogenesis [85,86]; hence, such homeostasis might be considered a primary target for new pharmacotherapies.

### 2.3. Which Came First: the Chicken or the Egg? “Dendrimer-Glucose” *vs.* “Dendrimer-Protein” Interactions Underlying the Anti-Hyperglycaemic Effect of PAMAM Dendrimers

The occurrence of oxidative and glycoxidative stress is postulated to be the principal event in the pathogenesis of diabetes mellitus and its complications. Therefore, AGEs and AOPPs (advanced oxidative protein products), which are specific end-products derived from non-enzymatic reactions, are considered potentially useful biomarkers for this disease. In particular, AGEs are represented by a heterogeneous group of bioactive compounds (e.g., pentosidine, carboxymethyllysine and imidazolone) that are formed by the nonenzymatic glycosylation of biomacromolecules. Some AGEs demonstrate characteristic fluorescence and are able to crosslink protein molecules and to bind to AGE-specific receptors such as RAGE [87]. AOPPs comprise various proteins, predominantly albumin and its aggregates that have been damaged by oxidative stress. AOPPs display several biological features that make them similar to AGEs. In particular, AOPPs are considered pro-inflammatory mediators that can damage biological membranes, affect the endothelium and impair HDL metabolism; thus, they are potential key players in the development of cardiovascular disease and immune dysregulation [88]. Glycation and oxidative stress resulting from hyperglycaemia and dyslipidaemia lead to accelerated non-enzymatic modification of essential biomacromolecules, particularly proteins. The nonenzymatic reaction between proteins and sugars (mainly glucose and fructose) leads to the formation of glycated proteins, which, depending on the number of glucose molecules condensed on the protein and the stereochemistry of the protein, are likely to exhibit functionalities that are different from those of native molecules.

Serum albumin is the most abundant circulating protein modified by early and advanced glycation, and glycated albumin (often recorded as fructosamine) is a well recognised marker that is associated with a high prevalence of macrovascular disease in diabetes mellitus. In fact, the presence of glycated albumin in plasma has been considered a more accurate predictor of diabetes complications than the routinely utilised glycated haemoglobin (HbA_1c_) [89]. Glycation also occurs in non-diabetic subjects, in whom, however, only up to 6% of haemoglobin and 12%–16% of serum albumin becomes glycated. The mechanisms leading to protein glycation in the non-diabetic state have not yet been unambiguously established. There are very few reported *in vitro* studies in which physiological concentrations of glucose have been used, and the results of these studies are inconclusive as to whether glucose alone can successfully promote glycation. In diabetic subjects, protein glycation is assumed to be an effect of mass action driven by high glucose concentrations. However, the complete process of AGE production involves both nonoxidative and oxidative pathways [90].

Non-enzymatic glycation is one of the underlying factors that contributes to various alterations in the intrinsic functions of proteins. It is a result of the covalent binding of glucose to amino groups of circulating proteins such as haemoglobin (HbA_1c_) and albumin or of proteins present in the extracellular matrix (such as collagen). Because of its relatively long half-life compared to other proteins (approximately 21 days) and its high concentration in blood, serum albumin belongs to a group of proteins that are highly vulnerable to glycation. Elevated levels of glycated albumin in diabetic individuals, representing a two- to threefold increase, may contribute to the development of irreversible tissue damage associated with metabolic disorders that are observed in pathologies such as retinopathy, nephropathy, neuropathy and coronary artery disease [91]. For this reason, the struggle with this diabetes-associated burden represents a real challenge in the treatment of late diabetic sequelae. The pharmacological protection of proteins against the detrimental effects of excessive glucose constitutes one of the milestones of such a struggle.

The idea of our study was simple; it was aimed at checking the possibility that cationic PAMAM dendrimers are able to scavenge excess free glucose present in an *in vitro* sample or in a living organism. The presence of excess free glucose underlies the mechanism of the process of non-enzymatic glycosylation (N-glycosylation, glycation), which occurs in the course of a reaction commonly known as the Maillard reaction cascade. This modification results from the covalent binding of glucose to amino groups of biomacromolecules [90]. It was proposed that chemical competition for accessible glucose between free amino groups in proteins and those on the PAMAM surface would help reduce the extent of unwanted protein modifications. Under these conditions, the “glucose attack” would be shifted towards the dendrimer’s primary amino groups instead of the protein’s amino groups. Therefore, our investigations, which were conducted under both *in vitro* and *in vivo* conditions, attempted to identify protein modifications that take place upon exposure to glucose in the presence of the tested dendrimers. The body of evidence accumulated since then has unambiguously shown that amine-terminated PAMAM dendrimers, regardless of the employed generation (G2, G3 or G4), contribute to considerably reduced protein glycation in a model of experimental diabetes. Thus, based on the levels of markers of diabetic complications such as AGEs, AOPPs and glycated haemoglobin, exposure to dendrimers protected proteins against the impact of glucose, as expected.

**Figure 15:**
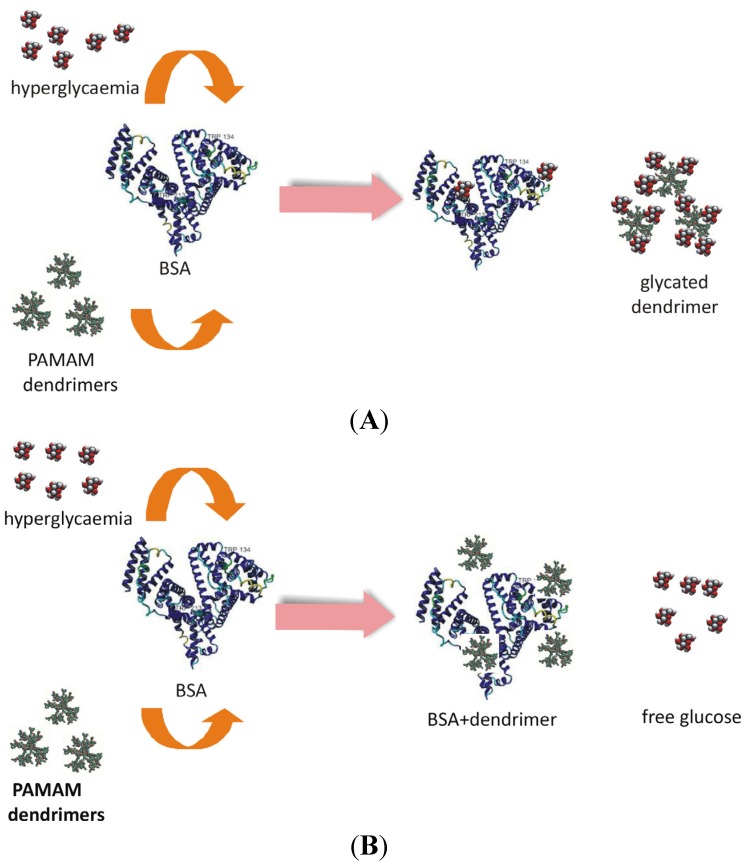
Two hypothetical pathways of PAMAM dendrimer interactions with albumin that could result in reduced albumin glycation. (**A**) The attachment of glucose to albumin free primary amino groups (albumin glycation) is hampered by PAMAM dendrimers, which scavenge the glucose and undergo glycation themselves. (**B**) PAMAM dendrimers interact with albumin molecules, which thereby become less susceptible to glycation.

Nevertheless, the data suggesting that PAMAMs have anti-hyperglycaemic effects leave the fundamental emerging question still open: do dendrimers ‘trap’ the excessive glucose and consequently compete for it with proteins, thus contributing to much reduced protein glycation, or do dendrimers directly interact with proteins and consequently make the covalent binding of glucose to protein amino groups unfavourable? Theoretically, a reduction in the number of glucose-occupied amino groups in glycated proteins may be observed in both cases, but the final “stoichiometric” outcome of these “reactions” is expected to differ. In the first case, the dendrimers are more likely to have a positive influence, while in the second case they would be expected to exert negative effects on the structure and function of proteins. Alternatively, should the proteins become impaired due to the dendrimer treatment, the protective action of dendrimers might be partially or completely lost. Finally, both pathways may also co-exist in a hypothetical experimental system (Figure 15).

Extensive studies on the interactions between dendrimers and proteins conducted in the early 2000s showed that the effects of interactions between cationic PAMAM dendrimers and proteins strongly depend not only on the dendrimer’s concentration, its chemical structure and its generation number [49,92] but also on environmental conditions such as pH and ionic strength (NaCl concentration) [93,94]. Importantly, it has been shown that the nature of the interactions between dendrimers and proteins may vary accordingly; this, of course, may affect the potential use of dendrimers in “hypoglycaemising” and/or anti-diabetic therapy. Strong and irreversible interactions between PAMAM dendrimers and proteins are likely to block or disturb the biological and physiological functions of these proteins. Adverse and undesirable interactions may lead to the loss of the natural protection in living organisms, e.g., against toxins [95]. If this occurs, it may be easily deduced that such interactions of dendrimers with proteins could be a factor that limits their use in medicine. For instance, PAMAM dendrimers have been recently shown to form complexes with proteins as well as to change the secondary structures of protein molecules [96]. PAMAM G4 dendrimers have also been shown to induce conformational changes and alter the electrostatic nature of bovine serum albumin (BSA) [49], an observation consistent with those of other studies that suggest the existence of strong dendrimer-protein interactions that are even likely to induce partial protein unfolding at high dendrimer concentrations [97]. Moreover, charged cationic PAMAM dendrimers have been demonstrated to destabilise proteins due to cooperative binding of the dendrimer surface groups to the protein surface or even to bind specifically to protein-protein interfaces, thereby inhibiting protein oligomerisation. Altogether, under such circumstances, the strong avidity observed in protein-dendrimer interactions may easily have negative effects on protein thermostability [98].

Our own findings obtained in an *in vitro* study [7] do not, however, concur with the reports mentioned above. In our study, we found neither significant binding of dendrimers to bovine serum albumin (BSA) after testing in various buffers, nor did we detect any effects of dendrimers on BSA conformation. Our data clearly indicate that various full-generation PAMAMs (a) do not interact with BSA; (b) do not affect protein conformation; and (c) do not form stable complexes with BSA at the concentrations used (G2: 120 µM and G4: 30 µM). Additionally, the *in vitro* glycated forms of dendrimers have not shown considerable interactions with BSA. One possible explanation for this phenomenon is that at the low concentrations of PAMAMs used in the above-mentioned study these dendrimers had no detectable impact on BSA structure [7]. Notably, however, although the studied PAMAM concentrations were relatively low, the dendrimers remained effective in competing with BSA for glucose molecules.

A valuable incentive for discussion of these observations was provided by the outcome of a study by Shcharbin *et al.* based on the crystallographic 3D analysis of HSA and BSA. These authors provided evidence that PAMAM dendrimers of the 3rd and higher generations are quite large (>3 nm) in comparison with the studied proteins (14 nm). Hence, the number of sites for the possible “docking” of PAMAMs at the surfaces of these proteins is certainly limited. These authors also noted that the interactions between BSA/HSA and PAMAM dendrimers are quite weak in polar solvents at neutral pH, making such interactions difficult to detect [99]. These findings were confirmed by Giri and co-workers, who used a number of sophisticated techniques (^1^H-NMR, saturation transfer difference (STD) NMR and NMR diffusion-ordered spectroscopy (DOSY)) to gain insight into the mechanism of HSA binding to PAMAM dendrimers [100]. The results of these experiments clearly indicated that the interactions between HSA and dendrimers are in general of a weak nature. Although commonly accepted, such a recognition of the problem stands in contrast to a report based on simulations data, in which it was demonstrated that rather strong interactions between HSA and the inner shell protons and neighbouring amide groups of the dendrimers are theoretically possible [100]. Additional similar findings exist in the literature concerning the relationships between dendrimers and proteins. It seems obvious that cationic dendrimers are large macromolecules that possess rather densely distributed positive charges at their surfaces at neutral pH. On the other hand, two large protein biopolymers, BSA and HSA, bear net negative charges (−17 for BSA and −15 for HSA) [101], derived from their low isoelectric points (approximately 5.2 for HSA [101] and 4.82 for BSA [102]). It is worth noting that while both albumins possess regions with strong negative charges, they also bear positively charged pockets [103,104], which might drive some researchers to the misleading conclusion that albumins, as proteins with positively charged surfaces, and dendrimers repulse each other. Hence, weak or no interactions between dendrimers and albumin proteins may be observed. In fact, deprotonation of amino acids occurs in the albumin molecule at neutral pH [105], and this results in the protein acquiring an increased negative charge. Thus, dendrimers are very likely to interact with albumins, even more so at physiological pH, conditions under which PAMAM dendrimers affect proteins to the maximum degree [94]. It has been suggested that the interaction occurs mainly between dendrimer polar groups (NH_2_) and protein hydrophilic groups (C=O, CN, and NH) as well as with Trp-212 of BSA and Trp-214 of HSA, which are located inside the protein molecule and provide hydrophobic contacts [106]. Some authors have even described five preferential domains/sites in the albumin molecule and suggested the likelihood of interaction of PAMAM G4 with these domains [99]. Interestingly, it has also been shown that the binding of dendrimers to proteins and their amino acid components is promoted, when both electrostatic and hydrophobic interactions occur. Moreover, the level of protonation of the amino groups of dendrimers is primarily responsible for this binding process, *i.e.*, the higher the level of dendrimer protonation, the stronger the interaction of dendrimers with proteins [107]. Overall, however, the complex array of data accumulated hitherto is inconsistent at present in claiming that the possible interactions between dendrimers and proteins in (patho)physiological states are either of low significance under conditions mimicking physiologically achievable concentrations or of high significance under model conditions involving much higher dendrimer concentrations. The first option is challenging for researchers active in the field of the innovative biotechnology of dendrimeric compounds. The second option may seem devastating, at least at first sight, to the hope of using these agents in new areas of pharmaceutical research.

Another very intriguing aspect of the problem of the existing strong relationship between proteins and dendrimers was recently presented in a report published by Klajnert *et al.* The authors of this paper raised the idea of the strong interactions between dendrimers and some proteins as a peculiar defence mechanism at the level of the organism. The authors postulate that interactions with selected proteins, such as some abundant proteins in blood plasma, *i.e.*, albumin, are likely to compromise the overall toxicity of dendrimers by suppressing their interactions with other targets, thus making dendrimers less harmful for proteins and lipids of crucial importance in the cell interior and cell membranes [108].

The direct implication of the idea that interactions between PAMAM dendrimers and albumin are at most of a weak nature, if they are possible at all, at the assumed concentrations of PAMAMs and proteins is that any effects occurring at the dendrimer-glucose interface would be likely to dominate those occurring at the dendrimer-protein interface. Furthermore, if we accept the idea that these polymers do not interact strongly with proteins, we must also assume that they do not contribute significantly to protein damage or to alterations in protein function. If we assume that PAMAMs act as modulators of the non-enzymatic modification of biomacromolecules under pathological conditions, it must also be assumed that we can demonstrate a role of PAMAM dendrimers as protectors against protein overload by excessive glucose. Thus, a lack of undesired interactions between these two competitors for glucose binding suggests that dendrimers could function as potential useful inhibitors in long-term complications in diabetes.

While appreciating all of the exploratory enthusiasm of the literature findings on potential dendrimer applications, it seems crucial to investigate whether polyamidoamine dendrimers interact with proteins in the presence of glucose. Which of these two interactions, “dendrimer-protein” or “dendrimer-glucose”, has primacy is a question that is still open for debate, although our results suggest that PAMAM dendrimers can be designed as effective chemical competitors of proteins for non-enzymatic modification by glucose and other naturally electrophilic by-products [5,6,7,21].

### 2.4. Glucose Scavenging or Toxic Impairment of Hepatic Gluconeogenesis? The Possible Ambiguity of the Hypoglycaemising Effects of PAMAM Dendrimers Tracked by HbA_1c_ Raises Some Doubts

The leading hypothesis concerning the anti-glycating properties of PAMAM dendrimers, which is based on the results of both *in vitro* and *in vivo* studies, has been discussed in detail in earlier paragraphs of this paper and in the literature [5,7]. Above, we clearly stated the drawbacks of using PAMAM dendrimers in medical practice. These drawbacks include the possibility of serious adverse effects associated with reduced animal survival. With regard to the toxic effects of PAMAM dendrimers, it is necessary to consider at least two possible factors that may delay the announcement of PAMAM dendrimers as safe euglycaemising agents: hepatic accumulation of PAMAM dendrimers, which is followed by impaired glucose metabolism in hepatocytes, and red blood cell haemolysis. Considering these phenomena, at least one alternate mechanism exists that may explain the presence of lower glycaemia in animals with experimentally induced diabetes after treatment with PAMAM dendrimers. This alternate mechanism, we must admit, may not seem attractive at first glance.

**Figure 16:**
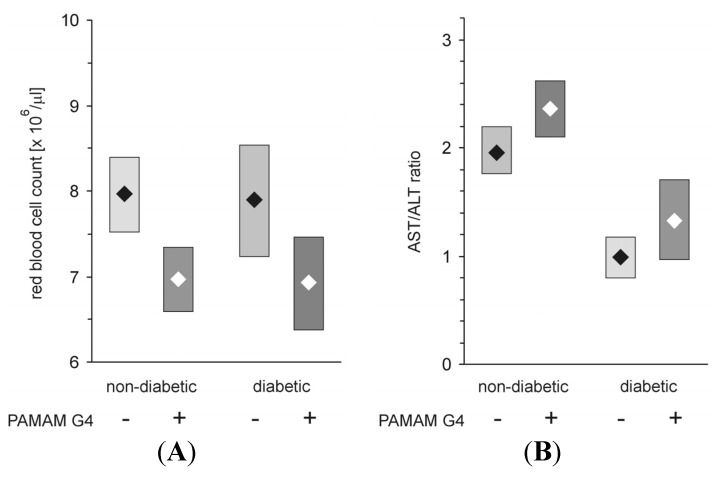
Effect of administration of PAMAM G4 on red blood cell count and aspartate aminotransferase/alanine aminotransferase activity ratio (AST/ALT ratio, De Ritis ratio) monitored in the peripheral blood of non-diabetic and streptozotocin-diabetic rats. The data are presented as the means (diamonds) with 95% CI (rectangles); n = 10–13 for streptozotocin-diabetic and 10–16 for non-diabetic Sprague-Dawley rats. The animals received PAMAM G4 intraperitoneally for 60 consecutive days. For details, see the text and earlier publications [5,19]. The significance of the differences between PAMAM-treated and control animals was: RBC count, *p* < 0.005 for non-diabetic and *p* < 0.05 for diabetic rats; AST/ALT ratio, *p* <0.02 for non-diabetic and *p* < 0.05 for diabetic animals.

A pivotal notion for further consideration is the pattern of PAMAM dendrimer biodistribution in the bodies of laboratory animals. It has been established that PAMAM dendrimers introduced into peripheral blood circulate for a relatively short period of time lasting approximately 1 hour. Almost 90% of injected amino-terminated, non-modified PAMAM dendrimer is retained in the liver [16], where its metabolic fate remains largely unknown. It is believed, however, that the putative effects exerted by PAMAMs, that have accumulated in the liver, may be quite similar to those that have been observed in other tissues exposed to dendrimers; these phenomena are generally referred to as concentration- and generation-dependent cytotoxicity of PAMAM dendrimers [16]. Because the liver is the primary site of glucose synthesis in mammals, it seems likely that toxic damage to hepatocytes might result in decreased levels of long-term glycaemic control markers such as glycated haemoglobin (HbA_1c_), *per analogiam* to the reduced HbA_1c_ concentrations observed, for instance, in patients with liver damage due to cirrhosis or chronic hepatitis [109]. The possible occurrence of hepatotoxic hypoglycaemia in animals treated with PAMAM dendrimers should be carefully tested, more so considering the fact that plasma biochemical measurements of hepatotoxicity markers, such as higher plasma activity of hepatic aminotransferases, are not always consistent. On this occasion, it should also be kept in mind that extreme reduction of glucose concentration is not always beneficial; one example is the immense reduction in glucose levels that are often observed in cases of insulinoma [110]. While our earlier results unambiguously spoke against PAMAM dendrimer-induced hepatotoxicity [5], our recent data suggests the opposite. We observed that one of the hallmarks of liver damage, the aspartate aminotransferase/alanine aminotransferase (AST/ALT) ratio (also known as the De Ritis ratio), was elevated in both streptozotocin-diabetic and non-diabetic animals treated with PAMAM G4, which tends to confirm the supposed hepatotoxicity of PAMAM dendrimers (Figure 16B). However, if PAMAM-induced hepatotoxicity-mediated hypoglycaemising effects occur, both fasting glucose and HbA_1c_ concentrations should be lowered. Greatly reduced values of HbA_1C_ were observed in diabetic animals treated with dendrimers; these values, which were within the normal [5] or almost-normal range [19], were in some disagreement with concomitantly recorded levels of blood glucose. Although blood glycaemia significantly decreased upon treatment of diabetic animals with PAMAM G4, it still remained within the hyperglycaemic range that is typical of “non-cured” diabetic animals. Such a disparity in HbA_1c_ levels and measured blood glucose levels might have resulted from the occurrence of toxic processes in animals treated with PAMAM dendrimers; such processes would be likely to influence HbA_1c_ measurements significantly and to have less pronounced effects on blood glycaemia. A few reactions characterised by a congruent pattern of modulation of blood glycaemia and HbA_1c_ concentration are described in a recent very interesting review by Hare and co-workers [111].

**Figure 17:**
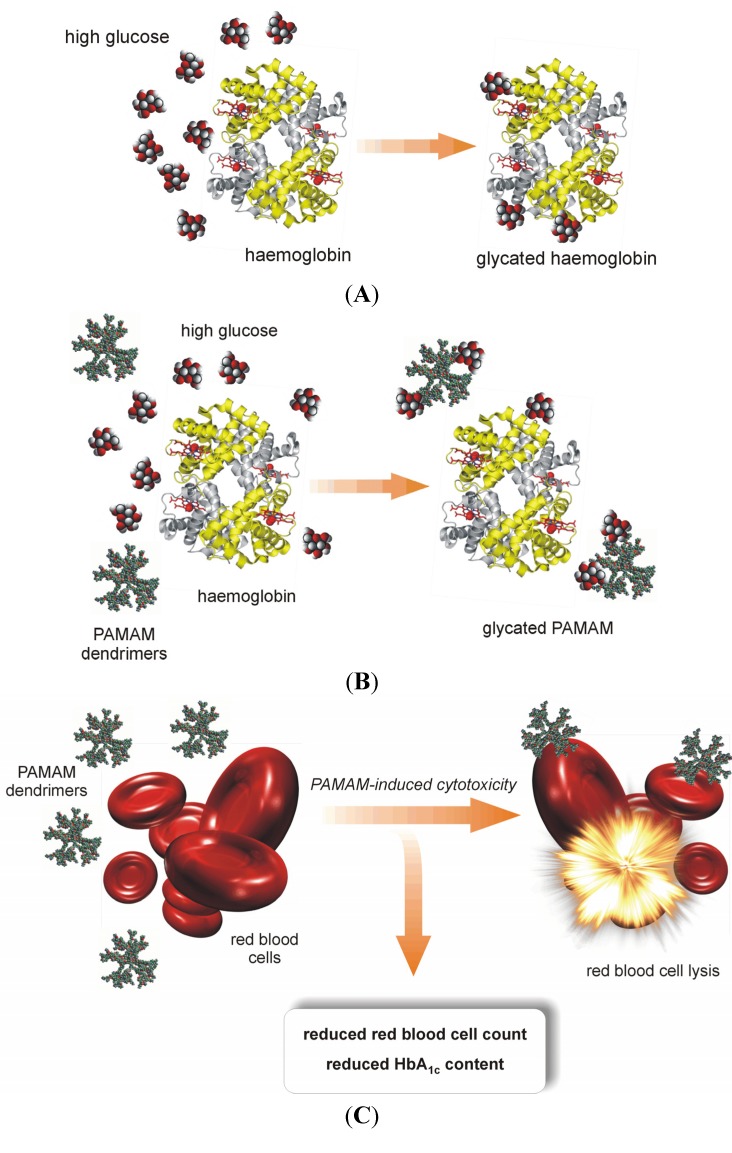
Two hypothetical mechanisms for the lowering of glycated haemoglobin content (HbA_1c_) by PAMAM dendrimers. Excessive glucose leads to increased non-enzymatic glycosylation (glycation) of haemoglobin (**A**), while scavenging of excess glucose by PAMAM dendrimers leads to a lowered rate of haemoglobin glycation (**B**). PAMAM-induced red blood cell haemolysis results in the presence of fewer haemoglobin target molecules, thus reducing the overall extent of non-enzymatic glycosylation of haemoglobin (**C**). Both pathways may result in a decrease in the fraction of haemoglobin molecules that are glycated in animals treated with PAMAM dendrimers.

The most likely toxic effects of PAMAM dendrimer treatment on rats may be the occurrence of advanced haemolysis, which shortens erythrocyte lifespan; in fact, dendrimer concentration- and generation-dependent haemolysis has been observed under *in vitro* conditions [112]. The possible lysis and degradation of red blood cells, the carriers of HbA_1c_, might of course theoretically contribute to artefactually lowered outcomes of HbA_1c_ determinations (Figure 17). The relevant question, however, is whether such dendrimer-induced haemolysis actually occurs and whether it affects HbA_1c_ levels in the peripheral blood of living organisms. Needless to say, circulating blood is a body fluid abundant in a variety of proteins that may act as potential dendrimer scavengers and thus also as antihaemolytic agents [113]. Only very occasionally has such haemolysis been monitored, and on those rare occasions it was not evidenced in living organisms treated with PAMAM dendrimers [5].

However, in a recent study, we found significant reductions in red blood cell count in Sprague-Dawley rats treated chronically with PAMAM G4, and this reduction occurred in both streptozotocin-diabetic and non-diabetic animals (Figure 16A).

Overall, at present we can not unambiguously conclude on the mechanisms leading to PAMAM-mediated reduction in red blood cell count. Haemolysis is probable, however, impaired iron metabolism or bone marrow toxicity should be also considered.

## 3. Concluding Remarks—the End of the Beginning or the Beginning of the End?

This paper presents our views concerning the use of full-generation PAMAM dendrimers in the area of diabetic care. We have shown that these compounds have the potential to scavenge excess glucose both *in vitro* and *in vivo*. Several studies have unambiguously demonstrated the potential of PAMAM dendrimers to reduce the levels of markers of long-lasting hyperglycaemia in living organisms. It is clear from the present overview that amine-terminated PAMAMs possess several of the required properties for good candidates for the therapy of diabetic hyperglycaemia.

On the other hand, we have demonstrated in several independent studies that daily treatment with these compounds can be dangerous and life-threatening. In our opinion, the dangers associated with long-term frequent injection of dendrimers seriously limit the use of these polymers in the treatment of diabetes. In preclinical investigations, the greatest problem may be the assessment of an appropriate dosage. As we have concluded from our observations, dendrimers of the same generation administered at one selected dose can have different effects depending on the season (autumn/winter *vs.* spring/summer) or on the rodent strain/breed used (e.g., Wistar *vs.* Sprague-Dawley rats) [21,44]. These findings suggest that dendrimer activity may be entirely predictable only when laboratory conditions are well characterised and when both the origin and the characteristics of the animals used are kept constant. Although a wide range of literature describing the interactions of PAMAMs with biological material is available, the majority of experimental results involving these compounds have been obtained with cell lines or in other *in vitro* studies. Therefore, further investigations are certainly needed to define a precise risk assessment for the use of PAMAM dendrimers in clinical applications provided, of course, that more benefits than disadvantages are found to be associated with dendrimer administration.

**Figure 18:**
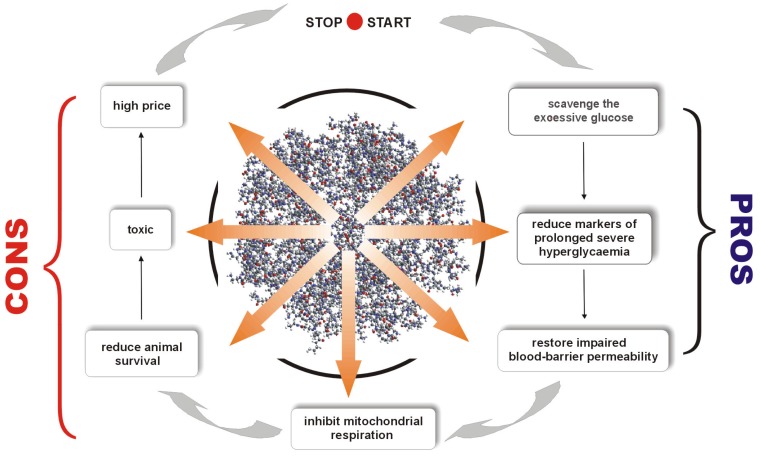
Advantages and disadvantages of using PAMAM dendrimers in the treatment of severe diabetic hyperglycaemia.

In our studies, particular emphasis was placed on the evaluation of dendrimer activity and toxicity, two issues that are of paramount importance for therapeutic use. It was demonstrated that cationic PAMAM dendrimers possess many desirable properties, including high effectiveness in the scavenging of excessive glucose and protective activity against the detrimental effects of long-lasting hyperglycaemia on proteins. Nevertheless, the evidence for the occurrence of serious detrimental effects when these compounds are used to treat animals with experimental diabetes cannot be ignored. To reinforce the above opinions concerning the possibility of using PAMAM dendrimers in long-term anti-glycation therapies in diabetes, we present a scheme showing the disadvantages and benefits of using PAMAMs in this model of disease (Figure 18). Based on the considerations presented in this review, it may be that this subject has gone full circle. Nevertheless, our work has provided crucial insights into PAMAM activity in the rat model of experimental diabetes, and we have significantly extended existing knowledge concerning these compounds.

To the best of our knowledge, there are no previously published reports on the use of plain, unmodified PAMAMs *in vivo* as hypoglycaemising agents to limit the consequences of untreated diabetes. The studies performed in our laboratories were focused on achieving an understanding of the profile of *in vivo* PAMAM toxicity and defining the mechanisms of the anti-glycation action of PAMAM dendrimers. The primary challenges in these studies included determination of non-toxic doses of these compounds and of the necessary duration of dendrimer supplementation, the choice of solvent, the route of dendrimer administration and extrapolation of *in vitro* results to *in vivo* data. The primary goal of our investigations was to find the most appropriate dendrimer generation and the most effective dosing for anti-glycation therapy.

Now, after a few intensive years of studies in this field, it is clear that less has been positively resolved than was intended. First, the correspondence between the *in vitro* and the *in vivo* data with respect to both toxicity and bioavailability remains a real challenge. There are no established algorithms suitable for extrapolating non-toxic concentrations determined in the course of *in vitro* experiments to appropriate dosages under *in vivo* conditions. Therefore, when planning *in vivo* experiments, it was necessary to rely on the outcomes of other *in vivo* studies reported in the literature. Unfortunately, during the course of the experiments, the selected doses often proved to be too toxic for the tested animals. Another important issue is the use of methanol as a solvent in all reported animal studies on the effects of PAMAM dendrimers in severe hyperglycamia. Methanol was certainly an appropriate choice for studies in animals owing to the good stability of PAMAM dendrimers in this solvent. However, such a scheme may not be regarded as relevant to any possible therapy in humans because the targeting of a chronic disease excludes the use of methanol. This raises further questions on the stability of water solutions of PAMAM dendrimers when attempting to use similar treatments in humans. Additionally, the very high cost of dendrimers limits the ability to repeat survival experiments to find a balance between toxicity and effectiveness of PAMAMs at scavenging excess glucose. In our hands, such considerations had a considerable impact on the experimental design of our *in vivo* studies and seriously affected the conclusions of the studies, forcing us to leave many points unsatisfied and unfulfilled.

In our opinion, the most significant obstacle to the effective use of PAMAM dendrimers as anti-glycation agents is their uncontrolled toxicity and poorly established pharmacokinetics in living organisms. However, we believe that the majority of the adverse side effects caused by dendrimers can be limited and/or overcome by further research in this area. To summarise, the following issues must be addressed:

- overall PAMAM toxicity associated with the polycationicity of dendrimers at the organismic, organic and cellular levels;
- development of a reliable method for evaluating non-toxic/optimal doses of PAMAM dendrimers should be developed;
- determination of the optimal route of dendrimer administration and the choice of an optimal and non-toxic solvent;
- the significance of seasonality in monitoring of the *in vivo* effects;
- the problem of the relevance of *in vitro* and *in vivo* studies should be solved;
- the high cost of dendrimer production, which limits their application in chronic diseases (e.g., diabetes).
